# ESCDL-1, a new cell line derived from chicken embryonic stem cells, supports efficient replication of Mardiviruses

**DOI:** 10.1371/journal.pone.0175259

**Published:** 2017-04-13

**Authors:** Jean-François Vautherot, Christian Jean, Laetitia Fragnet-Trapp, Sylvie Rémy, Danièle Chabanne-Vautherot, Guillaume Montillet, Aurélie Fuet, Caroline Denesvre, Bertrand Pain

**Affiliations:** 1 ISP, INRA, Université François Rabelais de Tours, UMR 1282, Nouzilly, France; 2 Univ Lyon, Université Lyon 1, INSERM, INRA, Stem Cell and Brain Research Institute, U1208, USC1361, Bron, France; University of Minnesota College of Veterinary Medicine, UNITED STATES

## Abstract

Marek’s disease virus is the etiological agent of a major lymphoproliferative disorder in poultry and the prototype of the Mardivirus genus. Primary avian somatic cells are currently used for virus replication and vaccine production, but they are largely refractory to any genetic modification compatible with the preservation of intact viral susceptibility. We explored the concept of induction of viral replication permissiveness in an established pluripotent chicken embryonic stem cell-line (cES) in order to derive a new fully susceptible cell-line. Chicken ES cells were not permissive for Mardivirus infection, but as soon as differentiation was triggered, replication of Marek’s disease virus was detected. From a panel of cyto-differentiating agents, hexamethylene bis (acetamide) (HMBA) was found to be the most efficient regarding the induction of permissiveness. These initial findings prompted us to analyse the effect of HMBA on gene expression, to derive a new mesenchymal cell line, the so-called ESCDL-1, and monitor its susceptibility for Mardivirus replication. All Mardiviruses tested so far replicated equally well on primary embryonic skin cells and on ESCDL-1, and the latter showed no variation related to its passage number in its permissiveness for virus infection. Viral morphogenesis studies confirmed efficient multiplication with, as in other *in vitro* models, no extra-cellular virus production. We could show that ESCDL-1 can be transfected to express a transgene and subsequently cloned without any loss in permissiveness. Consequently, ESCDL-1 was genetically modified to complement viral gene deletions thus yielding stable trans-complementing cell lines. We herein claim that derivation of stable differentiated cell-lines from cES cell lines might be an alternative solution to the cultivation of primary cells for virology studies.

## Introduction

Marek’s disease (MD), a fatal T cell lymphoma in chickens is caused by an alphaherpesvirus, Marek’s Disease Virus (MDV, Gallid Herpesvirus 2—GaHV-2), and remains one of the major viral diseases affecting poultry production [1]. Marek’s disease virus is the prototype species of the Mardivirus genus within the subfamily of *Alphaherpesvirinae* [2]. The Mardivirus genus encompasses GaHV-2, the non-oncogenic Gallid Herpesvirus 3 (GaHV-3) and Meleagrid Herpesvirus 1 (MeHV-1—HVT), both found in gallinaceans, together with the Columbid Herpesvirus 1 (CoHV-1) and the Anatid Herpesvirus 1, respectively affecting columbids and their predators [3] and waterfowl [4]. Mardiviruses are host restricted, affecting birds only and replicating only in avian cells. During the course of the MD, GaHV-2 replicates in a variety of cells of the lymphoid, mesenchymal and epithelial/epidermal lineages within its host, but the virus appears to be highly cell-associated, spreading to uninfected tissues in a cell-to-cell manner. Dissemination of the virus from bird to bird is made possible by the release of infectious material from the infected feather follicle epithelium (FFE) [5, 6]. *In vitro*, GaHV-2 and -3 may be cultivated in primary cells of diverse origin such as embryonic fibroblasts from chicken and ducks (CEF or DEF) [7] or chicken kidney cells (CKC) [8], but in all cells the infectivity remains cell-associated and no extracellular virion production has been identified [9]. For research prospects, the major drawback of primary cells lies in the difficulty to dissect viral replication pathways by regulating viral or cellular genes of interest. Indeed creation of stable cell lines retaining permissiveness to GaHV-2 with up- or down-regulated genes remains a challenging objective. A limited number of permanent cell-lines supporting GaHV-2 replication have been described, some being less permissive than primary cells [10, 11], and some even contaminated by MDV [12, 13]. Interestingly, SOgE cells, derived from QM7 cell-line expressing GaHV-2 glycoprotein E, were described to support the replication of virulent, as well as cell-adapted vaccine viruses [14], and another cell-line, JBJ-1 is currently under study for vaccine production with cell-adapted vaccine Mardiviruses [15]. As experiments designed to generate new cell lines from primary cells [10], some combined with chemical treatment [10, 11], yielded disappointing results regarding the overall susceptibility of these cells for Mardiviruses, we hypothesized that a rationale for building a better cell-line could be based on the use of embryonic stem (ES) cells. Pluripotent ES cells have been derived initially from mouse and then from several animal species, [16] including chickens [17]. There has been a growing interest for the use of stem cells in virology as they can be induced to differentiate towards lineages that are difficult to obtain from primary cells or to establish as long term proliferating cell lines. When oriented toward a defined lineage, the differentiation of ES cells may lead to a unique permissiveness associated with a highly differentiated state as described for hepatocyte/hepatitis C virus interaction [18]. In this respect we have derived keratinocytes from cES cells [19] and shown that they were susceptible to GaHV-2 replication [20]. In addition to the strategy of lineage-orientated differentiation, we tested the idea that differentiating agents with multiple effects could drive cES cell differentiation toward an increased susceptibility to Mardiviruses. As reported for HHV-3 on hES cells [21], we found that cES cells were refractory to productive GaHV-2 infection. We hypothesized that Mardivirus permissiveness was dependent on the differentiation of the cells and that it could be monitored *in vitro* by inducing the pluripotent cES cells to differentiate. We first examined the conditions in which cES cells could be rendered permissive to GaHV-2 infection by using cyto-differentiating drugs and found that *N*,*N′*-hexamethylene bis(acetamide) (HMBA) was the most promising. Next, we analysed the transcriptomic profile between permissive and non-permissive cells leading to the potential identification of candidate genes that could be involved in cell permissiveness. Finally, we derived the ESCDL-1 line from cES cells by combining stromal and chemical (HMBA) induction and characterized this newly established mesenchymal cell-line by deep RNA sequencing. Mardiviruses of the GaHV-2 and MeHV-1 genotypes readily infected ESCDL-1 cells, and virus titres were in the range of those obtained on primary cells. ESCDL-1 have been serially passaged more than 80 times, meanwhile keeping their unique phenotypic features and being transfected, subcloned and used as complementing cell-lines without loosing their permissiveness. Therefore, the newly established ESCDL-1 cell line represents an attractive alternative to the tedious preparation of primary cells for research on Mardiviruses.

## Materials and methods

### Growth factors, chemicals and antibodies

Growth factors and cytokines (Peprotech, France) for cES cells growth were used as described [22]. HMBA [*N*,*N′*-Hexamethylene bis(acétamide)], ATRA (all trans-retinoïc acid), Sodium Valproate, TSA (Trichostatine A) and DMSO (Dimethyl sulfoxyde) were purchased from Sigma Aldrich and used as indicated in the text.

Monoclonal antibodies (Mabs) to GaHV-2 VP22, gB, ICP4 have been described [23, 24]. Mabs against pUL37 (S1A Fig), gE and gI were obtained by using baculovirus expressed antigens for immunization as described [23]. The monoclonal antibodies against avian mitochondria (4C7/2E4) [25], vimentin (AMF-17b) [26], cytokeratin type II (1h5) and actin (JLA20) [27] were obtained from the Developmental Studies Hybridoma Bank, created by the NICHD of the NIH and maintained at The University of Iowa, Department of Biology, Iowa City, IA 52242. The monoclonal antibody 11E10 to cytokeratin type I has been described [20]. The monoclonal antibody 5F8 (Chromo Tek GmbH) was used to visualize the viral protein pUL17 fused in frame with mRFP. The polyclonal hyperimmune chicken serum against MDV was kindly given by Dr Sascha Trapp.

### Viruses

Parental and recombinant GaHV-2 were derived using either the bacterial artificial chromosome (BAC) BAC20 [28] or the BACRB-1B from the original pRB-1B-5 BAC [29]. The BAC20ΔUL49 [30], BAC20EGFPVP22 [9] and BAC20UL17mRFP [31] have been described and were used to produce the corresponding viruses by transfection either in CESC or in ESCDL-1. Mutant BACRB-1BUL17mRFP was generated from the BAC clone of the RB-1B strain [32] by using the primers described previously [31]. A deletion affecting the UL37 gene was introduced into the BACRB-1B backbone. Deletion of UL37 ORF (ΔUL37) was performed essentially as described [31] using Del37F (5’-TATCGATCGCATCTAGGGAGAACAGAGGGTTGGCGATAGTTGCCTGAATAgtgtaggctggagctgcttcg-3’) and Del37R (5’-TCCAAGACGGGACCGTTGACATAAACGCCCGGATGTGGAGAATAGATTTCcatatgaatatcctcctta-3’) primers, with MDV specific sequences shown in capital letters. BACs were purified by standard anion exchange techniques (Macherey Nagel—France) and characterized by RFLP analyses and southern blotting (S1B Fig).

Meleagrid herpesvirus 1 (MeHV-1), strain FC-126, was reconstituted from a lyophilised vaccine preparation (Lyomarex^®^—Mérial) and serially passaged either on primary cells. Mardivirus inocula consisted in infected cells trypsinized when cytopathogenic effect (CPE) was maximal, cryopreserved in 95% foetal bovine serum (FBS)/5% DMSO and stored in liquid nitrogen.

GaHV-1 (ILTV) was also reconstituted from the lyophilised vaccine preparation Nobilis^®^ (Intervet) and passaged on LMH cells as described [33]. In the case of GaHV-1, the inocula were supernatant of infected LMH cells, collected at 4 to 5 day post-infection (pi), centrifuged at 200 × g to remove cell debris, aliquoted and stored at -80°C.

### Plasmids

Plasmid pCDNA3-UL49 has been described [23]. Plasmid pCDNA3-UL37 was constructed by cloning the coding sequence of the UL37 gene from GaHV-2 (Strain RB-1B) after amplification by primers UL37F (aggcctATGTCTGCCGTAACGACCGA) and UL37R (ctgcagTTATGCATTATCACCGTTTG) primers with *Stu*I and *Pst*I sites underlined and MDV UL37 sequence in capital letters. The UL37 ORF sequence was first cloned in pGEM T Easy, sequenced (MWG, France) and then cloned, either in the transfer plasmid pFastBAC for baculovirus expression or in pCDNA3 for expression under the minimal CMV promoter.

### Cells and culture conditions

Primary chicken embryo skin cell (CESC) cultures have been described [23]. The cES cells were maintained as described [17, 22]. The DF1 (ATCC –CRL 12203) and the LMH cells (ATCC-CRL 21117) were maintained in DMEM/F12 medium containing 10% FBS, 1% Penicillin Streptomycin stock solution.

Telomerase assay [34], cell-cycle analyses [35] and cell replication curves were performed as described.

### cES cells differentiation

cES cells were seeded at 1x10^5^ cell/cm^2^ into gelatin (Sigma) coated culture dishes in William’s E (WE) medium (Lonza) with 10% FBS. After one day, the concentration of FBS was reduced to 2% and chicken serum (CS) (Gibco—Invitrogen) added to a concentration of 3%, in the same medium. Confluent monolayers were obtained 3 to 4 days after and exposed for 24 to 48 hours to the differentiating drugs in WE with 1% FBS and 1.5% CS. After the differentiating pulse, the cells were infected as described below.

For the derivation of ESCDL-1, cES cells were seeded at a density of 2 x10^4^ /cm^2^ in flasks containing an extracellular matrix from primary CESC, prepared as described [36]. Cells were initially cultured in WE containing 10% FBS for 4 days after which the FBS concentration was reduced to 1% and 1.5% CS was added. The medium was changed once and, after 3 days, the cell monolayer was dissociated and seeded in a gelatin-coated flask in WE containing 10% FBS. After 4 days the cells were dissociated again and subcultured in WE with 10% FBS for 4 days (passage 2) and then for 1 day after a new dissociation (passage 3) before being exposed to 5mM HMBA in WE containing 1% FBS and 1.5% CS for 4 days. After the HMBA induction, the cells were dissociated (passage 4) and incubated in WE with 10% for 3 days after which the medium was replaced by WE with 1% FBS and 1.5% CS. Cells at passage 4 in serum reduced medium were kept for 15 days at 37°C after which they were trypsinized and further subcultured in DMEM-F12 containing 10% FBS for 3 additional serial passages (5 to 7). From passage 7 on, the cells were denominated ESCDL-1 (Embryonic Stem Cell Derived Line 1), cultured in DMEM-F12 with 10% FBS, and split twice a week at ratio of 1:3.

### Transfection

ESCDL-1 cells were transfected by using the Amaxa^™^ Nucleofector^™^ technology (Lonza—France) with the Basic Fibroblast kit (ref VPI-1002) and program F024. DNA amounts varied from 4 μg (plasmids) to 8 μg (BAC) for 4x10^6^ cells. After transfection, the cells were seeded in 2 gelatin-coated wells of a 6-well plate for 8 to 16 hours. The medium was then changed to either DMEM-F12 with 1.5% CS and 1% FBS after BAC transfection, or to selection medium when ESCDL-1 were transfected with plasmids coding for viral or reporter genes. Selection medium after transfection of pCDNA3 consisted in DMEM-F12 with 10% FBS and 1 mg/ml G418 (Sigma Aldrich). When needed, plasmid pTK-Hyg (Clonetech), coding for the hygromycine B phosphotransferase (Hph), was co-transfected at a ratio of 1 to 20 with the plasmid of interest and selection was performed with hygromycine B at 80 μg/ml (Sigma Aldrich). Cells at 24 h post transfection with Venus/pCS2 and pTKHYG were either directly exposed to hygromycin or trypsinized and seeded in a 78 cm^2^ Petri dish to be exposed to hygromycin selection one day later.

### Viral infections

To avoid any contamination of ESCDL-1 or differentiated cES cells by primary chicken cells, initial infection by avian herpesviruses was performed by using either transfection of BACs, FACS-sorted infected CESC [9] or cell-free viral inoculum (GaHV-1).

Infection with sorted infected cells was performed as described [37]. For GaHV-1 infections, lyophilised vaccine preparation was re-suspended in DMEM-F12 containing 1% FBS and 1.5% CS, diluted and inoculated onto LMH or ESCDL-1 monolayers according to standard protocols.

Mardivirus inocula from ESCDL-1 were prepared as described in the upper section.

Titration of Mardiviruses was performed as described [38], except that cell monolayers were overlaid by a semi-solid medium containing 1% methylcellulose (ref 25–449.182 VWR) in DMEM-F12 with 1% FBS and 1.5% CS. After 3 days (vBAC20 and mutants) or 4 days (vBACRB-1B and mutants) cell monolayers were fixed, permeabilized, and plaques were visualised by performing indirect immunofluorescence (IIF) as previously described [23]. Plaque counts were recorded from a minimum of 3 replicates per dilution and mean titres were expressed ± 2 standard deviations. Measurement of plaque areas and statistical analysis were performed as described [37] on a minimum of 80 plaques per experiment.

The GaHV-2 copy number in infected cells was quantified in a TaqMan real-time qPCR assay as described [32, 39] and the numbers of copies of ICP4 and cellular iNOS were calculated as described [40].

### Detection of viral and cellular antigens

Indirect immunofluorescence (IIF) was performed as described [31]. Secondary antibodies were either anti-mouse IgG, or anti-chicken IgG conjugated to Alexa Fluor^®^ 488 or 594 (Invitrogen—France). Polymerised actin (F-actin) was detected using Alexa Fluor^®^ 594 Phalloidin (Invitrogen- France) and cell nuclei were stained by Hoechst 33342 (Invitrogen—France). Preparations were viewed under a Zeiss Axiovert 200 M microscope, and de-blurred images or optical sections were obtained by using the Zeiss ApoTome structured illumination microscopy (SIM) system. Photographs were obtained with a Zeiss digital camera AxioCam MRm, using Axiovision software. Images were compiled for publication with Adobe Photoshop CS6.

### Western blot analysis

Cells were lysed in sample buffer and proteins were resolved on SDS-polyacrylamide gels [41], transferred to nitrocellulose membranes, and blots were processed essentially as described [31]. Bound antibodies were detected by using Supersignal West Pico Chemiluminescent substrate (Pierce protein biology products—Thermoscientific). Chemiluminescence signal was acquired using a Fusion FX7 system and images were processed with Adobe Photoshop CS6 software.

### Transmission electron microscopy

ESCDL-1 cells at passage 25 were grown in Nunc UpCell 6-well Multidishes (Nunc UpCell surface—Thermoscientific), infected with 2.9x10^4^ PFU per well of vBAC20 virus at passage 2 after transfection on ESCDL-1 or mock-infected. Monolayers were lifted from the dish as specified in the manufacturer’s instructions, immediately fixed and processed as described [9]. High-resolution images resulting from the merge of several contiguous images were obtained by using the Adobe Photoshop CS6 software.

### Microarray analysis

The cES cells and CESCs were subjected to RNA extraction after exposure to 5mM HMBA and infection by a cell-sorted inoculum of vBAC20UL49GFP, as described [9]. A biological replicate was produced for each condition, including the non-treated cells and the HMBA treated non-infected cells as controls, leading to the testing of 12 different independent samples. RNA preparation, labelling and microarray (4x44k GE chicken V2 slides (#G2519F-26441, Agilent)) hybridizations were performed as described [42]. Each of the 12 samples were randomly chosen and hybridized, resulting in a total of 12 hybridized pairs of samples. Raw data acquisition and analysis was also performed with a corrected p-value chosen as significant when < 0.05 [42]. The expression values and the identification of differentially expressed genes were done with R program and Limma library using a separate channel analysis of 2 color data. Differentially expressed genes were then filtered with log fold change ratios (Log(FC) between pairs of conditions of less than -2 and more than 2. Probes sequences from the filtered gene lists were submitted to the DAVID GO analysis tool (https://david.ncifcrf.gov/) and after the probe ID conversion, the GO analysis was performed with a p-value of 0.05 as a limit to define cluster of genes with predicted functions. The R package FactoMineR was used to perform PCA on all 44k probes using the NCBI GEO (http://www.ncbi.nlm.nih.gov/geo/) datasets GSE61221 for the CEF-1, GSE38168 for the CEF-2, GSE47191 for the DF1 cells.

### ESCDL1 RNAseq analysis

All the RNAseq analyses were performed by Helixio (http://www.helixio.com/). One μg of total RNA from ESCDL-1 at passage 31 from the initial cES cells was used to prepare the library using the TruSeq R Stranded mRNA sample preparation kit (Illumina). The average sizes, estimated by Bioanalyser (Agilent) and molarities, estimated by Qubit (Life Technologies) were of 268bp, 277 bp and 267 bp and of 157 nM, 202 nM and 94.5 nM for CEF, cES cells and ESCDL-1 respectively. The paired-end sequencing was performed on the NextSeq 500 sequencer (Illumina) and controlled by the SeqencingAnalysis viewer software (Illumina). The quality control of the ‘Sample-ID.fastq files was performed with the FasQC software (Babraham Institute). The total number of reads was of 85093694, 108943912 and 87951932 and the percentage of aligned reads reached 90.8, 82.9 and 82.2 for CEF, cES cells and ESCDL-1 respectively. The alignment was performed on the chicken genome (http://www.ensembl.org/Gallus_gallus/Info/Index, Galgal4) using the Hisat, subread and R (DEseq2) package [43]. The String software (http://string-db.org/) was used to identify putative networks between proteins in order to better define cellular phenotypes [44].

### QRT-PCR analysis

Quantitative real time PCR was performed using StepOne Plus TM Real Time PCR system (Applied Biosystems) as described [42]. All oligonucleotides (provided by Eurogentec) were designed with Primer 3 (S1 Table) from sequences extracted from the chicken genome (http://www.ensembl.org/Gallus_gallus/Info/Index, Galgal4). Two independent samples were run each in triplicate and the StepOne Plus TM software provides the RQ value for each one with the RSP17 (X07257) as internal control using the ΔΔCt method.

## Results

### Differentiation increases the permissiveness of cES cells for GaHV-2

**Fig 1:**
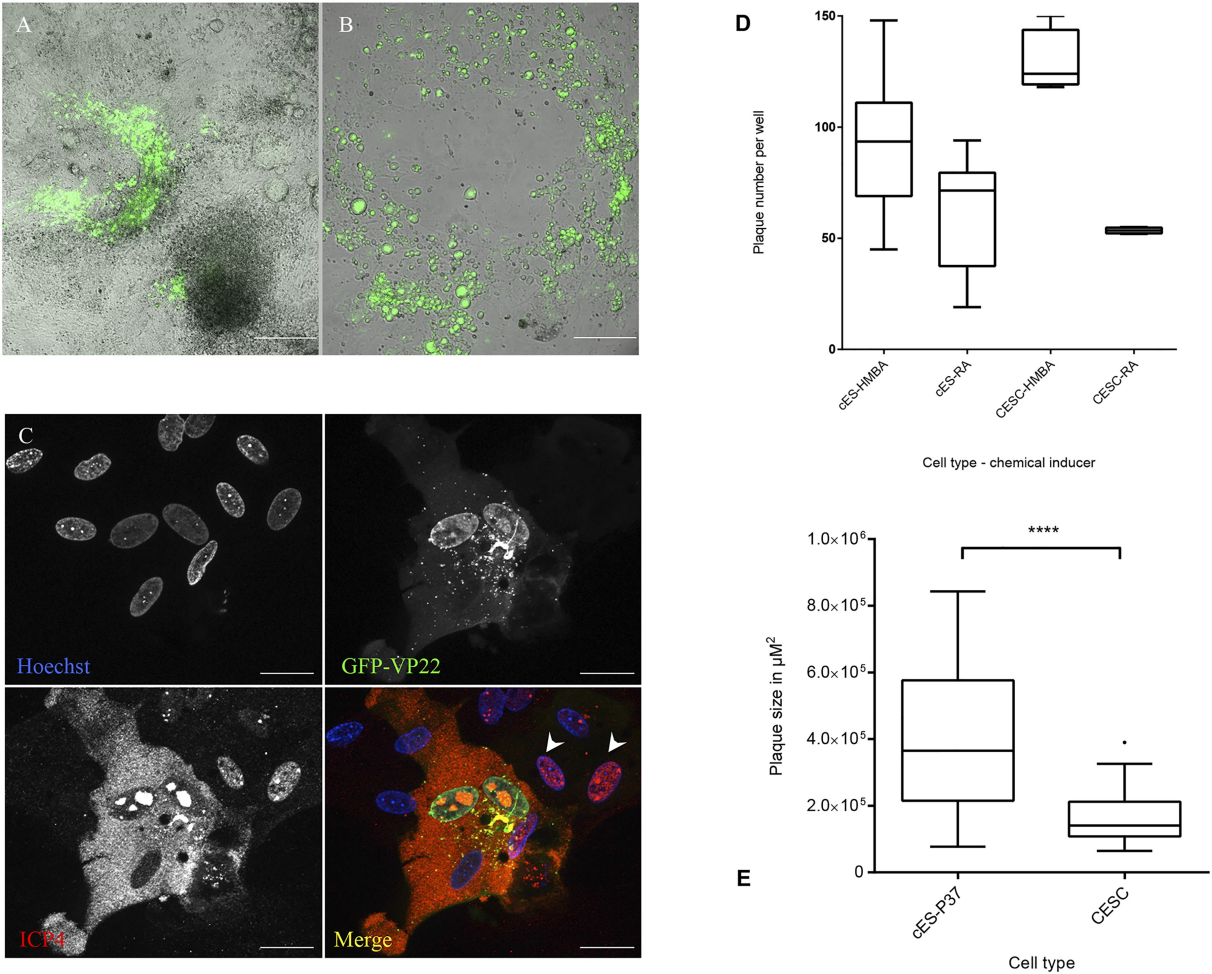
Differentiation increases permissiveness of cES to GaHV-2. (A & B): cES cells were plated and infected 6 days post plating in reduced serum conditions with sorted CESC infected with vBAC20GFPVP22. Cells were fixed after an incubation of 6 days at 37°C. (A) cell monolayers were maintained in WE medium containing 1% FBS and 1.5% CS. (B) DMSO (64 μM) was added from day 4 after plating and until the end of the culture (scale bar represents 200 μm). (C) cES cells (passage 36) were exposed to HMBA and infected with sorted vBAC20GFPVP22-infected cells. Expression of VP22 was detected by the GFP signal and ICP4 by staining with monoclonal antibody E21 (red); cell nuclei were stained by Hoechst 33342. At late stages of infection, ICP4 is detected both in the cytoplasm and nucleus in VP22 expressing cells. At early stages of infection, when VP22 is barely detectable in the cells surrounding the highly infected cell, ICP4 staining is predominantly nuclear (arrow heads) indicating spread of virus from the originally infected cell to the neighbouring cells (scale bar represents 20 μm). (D) Induction of differentiation by HMBA increases susceptibility of cES cells to GaHV-2 infection. Comparison of the plaque counts at 4 days pi either on cES cells or on primary CESC exposed to differentiating drugs (2 independent experiments sampling 10 replicates for each condition with cES and 4 replicates with CESC). (E) Comparison of plaque sizes on either cES exposed to HMBA differentiation or CESC. For both cell types, HMBA was added in the maintenance medium after the infection with sorted vBAC20EGFPVP22-infected cells. Plaques appeared larger in cES differentiated cells. Plaque sizes from 80 plaques per experiment are shown as boxplots and whiskers (Tukey) (in B, P<0.001; Mann Whitney test).

Initial tests performed to evaluate the level of replication of GaHV-2 in pluripotent cES cells showed that they were not susceptible to infection by the highly cell adapted vBAC20EGFPVP22, as concluded from the observation of infected monolayers at day 4 pi (data not shown). Infectivity evaluation assays included tests of media with reduced serum concentration, which induced differentiation in cES cells monolayers leading to cell morphological changes coinciding with an increase in permissiveness (Fig 1A). Bearing in mind that HMBA had a favourable effect on virus multiplication either for GaHV-2 [9] or for VZV [45], we tested a panel of cyto-differentiating drugs on cES cells plated on gelatin-coated surfaces. Over a 5 day period, the concentration of FBS was lowered from 10 to 1% with partial substitution by CS from 3 to 1.5%. This initial step primed cES cells differentiation, which was completed by exposing the cells to one of the following drugs, HMBA (5 mM), ATRA (1 μM), DMSO (64 μM), Valproate (300 mM), TSA (25 and 50 nM) or ascorbic acid (300 μM) together with CaCl_2_ (1.5 mM). After this differentiating pulse, cell monolayers were infected with vBAC20EGFPVP22 infected sorted cells. In all cyto-differentiating agents tested, HMBA, Ascorbic acid-CaCl_2_, DMSO (Fig 1B) and RA yielded interesting results regarding their differentiating activity, intrinsic toxicity and induction of permissiveness to GaHV-2. Viral plaques typical of GaHV-2 infection appeared at day 3 to 4 pi and staining for viral ICP4 early-late protein showed that viral infection spread from infected cells to the neighbouring ones (Fig 1C). Plating of a defined number of vBAC20EGFPVP22 infected sorted cells (13 000 cells per well in a 6-well plate) on either differentiated cells from cES or CESC showed that infection of the latter seemed to produce a higher number of plaques (Fig 1D), but the difference between cES differentiated cells and CESC was not significant when both cells were exposed to the same drug (Fig 1D). The areas of plaques were significantly larger in cES derived cells (Fig 1E). In this initial screening, HMBA appeared to have the most significant effect in driving cES differentiation towards permissiveness for MDV; however, direct differentiation from cES cells was cumbersome, with induction delays up to 5 or 6 days before infection, batch-to-batch variation and low virus production (2 x 10^3^ PFU/ml) compared to the standard CESC which consistently produced titres in the range of 5 x 10^5^ PFU/ml. We exploited this one shot induction approach to identify genes that were up- or down-regulated in response to HMBA exposure.

### The HMBA treatment modifies the transcriptomic profile of both CESCs and cES cells

In order to identify the genes or pathways that could be implicated in the cell permissiveness to GaHV-2, we performed a comparative microarray analysis on CESCs and cES cells, treated or non treated with HMBA, and either infected and non-infected. A total of 1092 and 548 probe IDs, in CESCs and cES, respectively, were found to be differentially expressed in HMBA treated versus non-treated cells. On virus infected HMBA treated cells, only 425 and 18 probe IDs were differentially expressed in the both type of cells (S2 Table). These IDs decreased from 1092 to 563, 548 to 266, 425 to 198 and 18 to 9 when unique gene symbol were used (S2 Table). Interestingly HMBA treatment seemed to exert a strong down-regulation on the transcriptome of either primary or cES cells as 900 (CESCs) and 389 (cES) genes were scored as down-regulated versus 192 (CESCs) and 159 (cES) scored as up-regulated (S2 Table).

**Fig 2:**
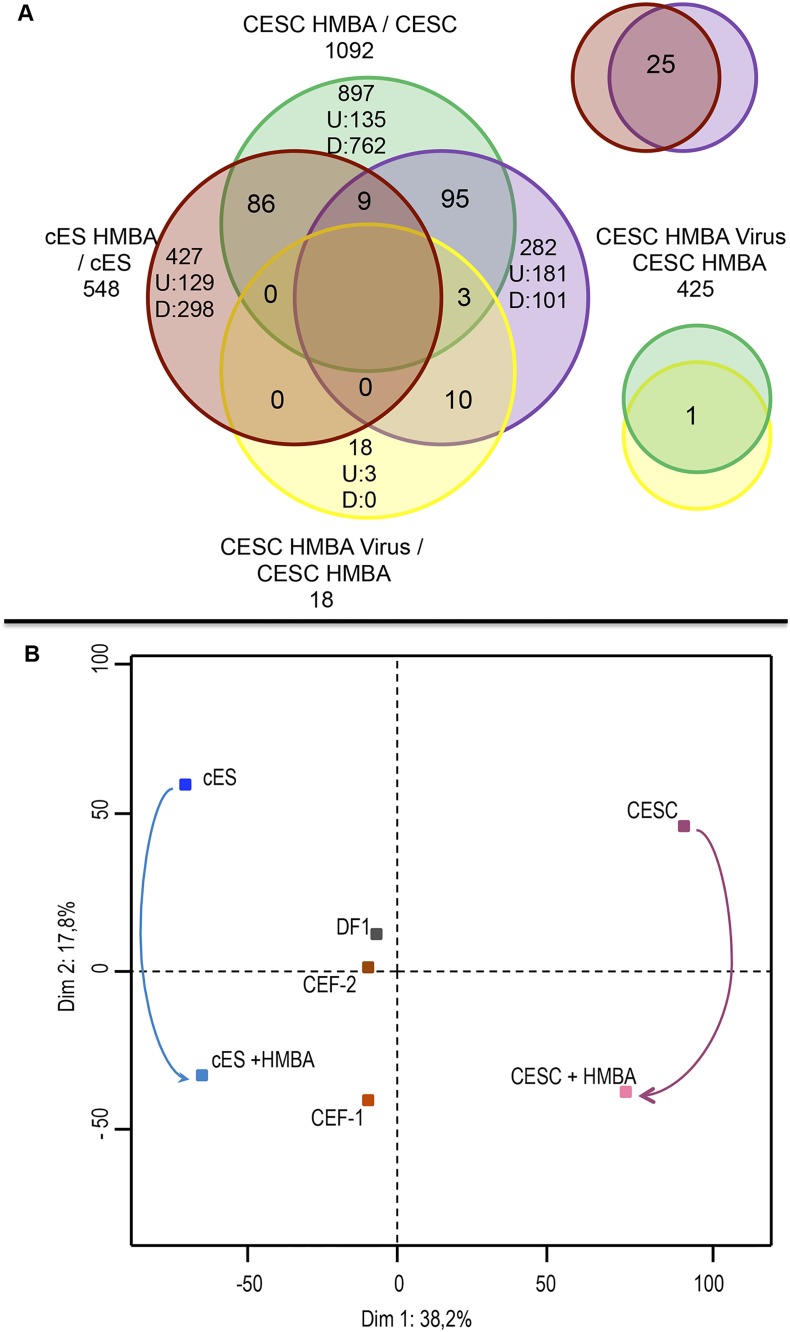
The HMBA treatment modifies the transcriptomic profile of both CESCs and cES cells. (A) The Venn diagram illustrates the genes that are differentially expressed between CESC and cES cells treated with or without HMBA and uninfected or infected with vBAC20UL49GFP inoculums. (B) A PCA analysis was performed on the common genes identified from this study and those provided by the datasets GSE61221 for the CEF-1, GSE38168 for the CEF-2 and GSE47191 for the DF1 cells.

The Venn diagram (Fig 2A) illustrates how genes are expressed in the different treated cells and outlines the higher number of genes differentially expressed in the CESC compared to the cES. In HMBA treated CESCs and cES cells, gene ontology (GO) analysis showed that up-regulated genes were associated with the Extracellular region and Microtubules-cytoskeleton for the Cellular components, Growth factor binding and Transferase activity for the Molecular function, and Regulation of biological process and Protein modification process for the Biological Process pathways (S1 File). For down-regulated genes in the same cells, again the Extracellular region pathways was identified, as well as lipid binding and cytokine activity for Molecular functions and of regulation of immune system process and of regulation of growth for Biological processes (S2 File). Those few pathways identified emphasize one of the main HMBA effects, which is change in cell morphology and phenotypic markers.

Using the datasets of primary fibroblasts (CEF) from two different origins, of DF1 fibroblastic cell line and of those CESCs and cES cells, treated and non-treated, a PCA analysis clearly established that the HMBA treatment resulted in a profile for the treated cES cells that resembled neither to CEF nor to DF1 cell line profiles (Fig 2B).

Interestingly, despite the very different origin of both cell types, when comparing both CESCs and cES treated versus non-treated cells, 96 probe ID (corresponding to 56 unique gene symbol) were found to be differentially expressed in both cell types, with more down-regulated (72) than up-regulated genes (18). Six genes (CDKN2A, TMP200C/LOC421054, ARAP3, LOC422654, CADPS and CR385173) were found to be up-regulated in treated cES cells, but down-regulated in treated CESCs cells, and Histone genes (LOC770022/HIST2H3L1, HIST1H46L4, HIST1H46L5), genes encoding transmembrane proteins (LOC421054/TMEM200C, TMEFF2, ASGP/MUC4), transferase proteins (MGAT3, HS6ST3) and few other ones (MT3, TECRG1L, LOC428714) were found to be up-regulated in both cell types (S2 File). No genes were found to be down-regulated in treated cES and up-regulated in CESCs treated cells.

### The MDV infection modifies the gene expression profile

Only 18 probe ID (corresponding to 11 gene ID) genes were up-regulated by the virus in cES cells compared with the HMBA treated cES cells when 425 were found (271 up and 154 down) for the CESCs cells in the same conditions (S2 File). Among the 11 genes, some are related to the immune response to a viral infection such as IRF1, IRF4, BATF3 and PELI2 [46]. Interestingly genes RASD1 [47] NEFL, CALCA and PIK3R5 are more involved in cell signalling and proliferation, possibly reflecting the impact of viral infection on cell-cycle. The TECPR2 gene appears to be a major actor in autophagy processes. More genes were found in the HMBA treated and infected CESCs, and a great number of them involved in the ‘regulation of immune system process’ according to the GO term of biological process.

### Derivation and characterization of ESCDL-1

**Fig 3:**
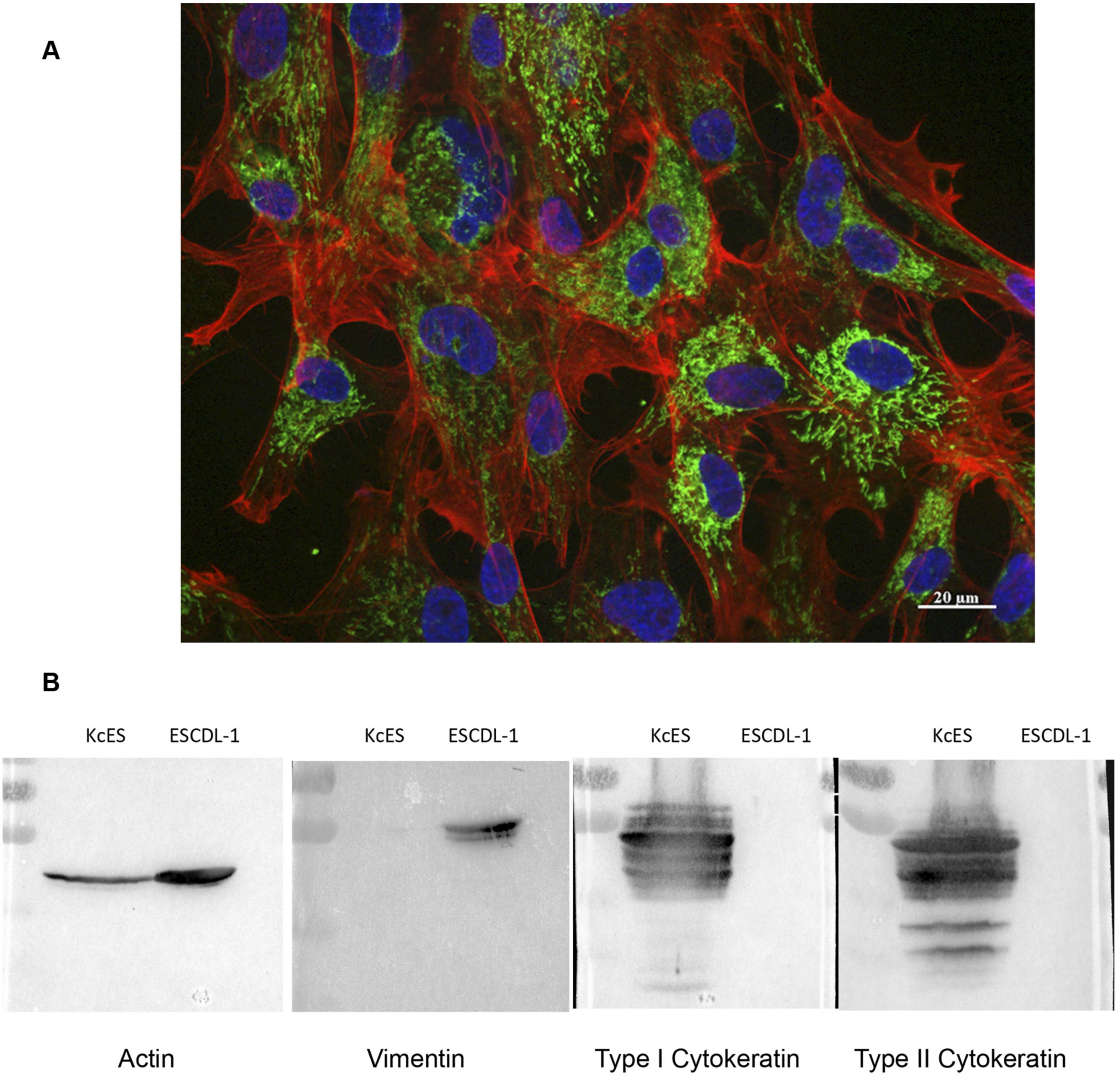
Characterization of ESCDL-1. (A) ESCDL-1 cells (passage 52) display a mesenchyme cell morphology with numerous actin stress fibres (Alexa Fluor^®^ 594 phalloidin). Nuclei appear in blue due to Hoechst 33342 staining, mitochondria in green by staining with monoclonal antibody 4C7 and Alexa Fluor^®^ 488 anti-mouse IgG. (B) ESCDL-1 express vimentin as a major intermediate filament protein. Proteins were extracted from chicken keratinocyte line K8 (KcES) and ESCDL-1 and western blots were probed with anti-actin JLA-20, anti-vimentin AMF17b, or anti-cytokeratin type I or II antibodies. The apparent molecular masses of actin (45 kDa) and vimentin (55 kDa) are similar to those described in the publications describing the monoclonal antibodies. ESCDL-1 cells do not express type I or II cytokeratins, which are detected in KcES extracts. Molecular weight markers (PageRuler^™^ Plus prestained protein ladder—Thermo Scientific) are on the left side of each blot.

The rationale for the derivation of a permissive cell-line from cES cells was to expose them successively to extra-cellular matrices (ECM) prepared from primary CESC {Stromal cell-Derived Inducing Activity—SDIA [48]} and to HMBA. For preparing ECM, the protocol described by Coreaux [36] was adapted to yield “a-cellular feeder matrices” from primary CESCs. These ECM preparations were stored up to 45 days at 4°C and tested for absence of living cells which could overgrow the cES-differentiating cells. At the end of the differentiation period, a new cell type emerged and was called ESCDL-1. These cells were then serially passaged in DMEM/F12 with 10% FBS, first once a week (passage 6 and 7) and then twice a week at a split ratio corresponding to the seeding of 3 to 4 x 10^4^ cells per cm^2^. The initial test for their susceptibility to GaHV-2 was performed on cells at passage 13 by using sorted cells infected with vBAC20 EGFPVP22vBAC20EGFPVP22, and proved to be satisfactory enough to further conduct ESCDL-1 characterization. ESCDL-1 cells displayed morphology of mesodermal cells with numerous actin stress fibers (Fig 3A) and strongly expressed vimentin, while no cytokeratins could be detected (Fig 3B). Telomerase activity in ESCDL-1 was equivalent to the telomerase activity of DF1 and CLEC cells [49] and much lower than the telomerase activity of the initial cES cells or of the transformed cell line LMH (S2A Fig). Cell cycle analysis of ESCDL-1 showed that ESCDL-1 profile was similar to primary cells indicating a robust growth for ESCDL-1 with approximately 20% of cells in S phase at 24 hours post plating (S2B Fig) and a rather strong contact-inhibition of replication (CIR) at 48h. This phenotype remained unchanged up to 100 serial passages (data not shown). The growth curve of ESCDL-1 showed a mean doubling time of approximately 40 hours, which appeared to be stable for at least 15 generations (S2C Fig).

The complete transcriptomic analysis of ESCDL1 was performed by deep RNA sequencing comparatively to the cES cells and primary embryonic fibroblasts (CEF) (S3 File). The acquisition of a differentiated phenotype included the loss of OCT4 (POU5F3), NANOG, SOX2, DNMT3B, LIN28B, CLDN1, ESRP2, ESRP1, EOMES and TRIM71, known markers of pluripotency for cES undifferentiated cells (42). Using the 574 genes differentially expressed by a FC > 5 between cES and ESCDL1, the first GO terms to characterize the ESCDL1 cells tended to define a cell with a strong extracellular matrix component with specific signalling pathways. However, as observed for the microarray analysis, on the 574 genes only 328 (less than 2/3) could be taken into account for the GO analysis, mainly due to the insufficient chicken genome annotation. No significant differences were found with a similar analysis performed with the 1172 gene differentially expressed by a FC > 4. The analysis of the protein-protein interactions of this first gene cluster (76 genes with an enrichment fold of 3.09 GO:0044421~extracellular region part), allowed the identification of a few nodes by using the String software. A first one was centred on IL-6 as a main cytokine linked to several growth factors, another one on CTGF in close relationship with a third one centred on SPARC, different collagens and the TIMP proteins, reinforcing the importance of extracellular matrix for the ESCDL-1 phenotype (S3A Fig). A unique set of integrins is expressed in ESCDL-1, being up-regulated when compared to the cES cells. The set included ITGA11, ITGA8, ITGAD (CD11d/CD18) and ITGA3 (CD49c) for the alpha-integrins and ITGB5, ITGBL1 and ITGB2 (CD18/MAC-1) for the beta-integrins. On the other hand expression of the ITGA6, more specific of a stem cell phenotype was found to be down regulated in ESCDL-1. We also observed an upregulation of THY1, NGF, VIM, PDGFRL and ENDOG genes, all highly expressed in mesenchymal stem cells. In good correlation with our findings on mesenchyme lineage markers in ESCDL-1 we also found a high expression of FOXC2/ MFH-1 (mesenchyme forkhead 1) gene, one of the three forkhead transcription factor (TF) highly expressed in mesenchymal stem cells, together with the CEBP (A, B and D) members (S3B Fig). Some of the genes modified by the HMBA treatment in both cES and CESC cells were also found to be strongly expressed in ESCDL1 such as the MGAT3 (FC = 4.6), WISP1 (FC = 7.63), IRF1 (FC = 2.5) (S4 Fig).

### ESCDL-1 are permissive to cell-adapted and “wild type” Mardiviruses

To monitor the susceptibility of ESCDL-1 to different GaHV-2, we used viruses that could be restored from corresponding BAC transfections in this cell-line. As it was shown previously that vBAC20UL17mRFP did not differ greatly from its parental vBAC20 virus in its replication (data not shown), we transfected the BAC20UL17mRFP (5.4 μg) in 4.5 x10^6^ of ESCDL-1 at passage 28 (Table 1). Viral plaques typical of MDV infection were visualised by day 4 post transfection and reached a total of 250 PFU on 2 wells in a six-well plate, 7 days after transfection. From this initial step (T1), vBAC20UL17mRFP was serially passaged and viral production over the first 12 passages was quantified by plaque titration (Table 1). Viral titres were in the range of 10^5^ from P3 on, occasionally reaching 10^6^ PFU/ml, and, when titrations were performed in parallel on CESC and ESCDL-1, no statistically significant differences could be observed.

**Table 1:** ESCDL-1 cells allow productive replication of vBAC20-UL17mRFP.

Passages	Inoculation conditions	Incubation time (days)	Cryovials/75cm2	Titres (PFU/ml)	
				CESC	ESCDL-1
**T1**^a^	5.4μg BAC/ 4.5x10^6^ ESCDL-1	7			250 PFU / 19 cm^2^
**P1**	7.8^b^	5	3	ND	ND
**P2**	5.5^b^	4	4	(3.4±0.3)x10^5^	(3.05±0.28)x10^5^
**P3**	0.003^c^	5	3	ND	(1.32±0.32)x10^5^
**P4**	0.01^c^	4	2.6	(1.1±0.95)x10^6^	(1.4±0.14)x10^6^
**P5**	0.1^c^	4	3	ND	(2.11±0.25)x10^5^
**P6**	0.015^c^	5	3	(2.17±0.24)x10^5^	(1.8±0.22)x10^5^
**P7**	0.01^c^	5	2.6	(5.67±1)x10^4^	(5.07±0.47)x10^4^
**P8**	0.002^c^	8	3.4	(3.9±0.26)x10^5^	(3.8±0.27)x10^5^
**P9b**	0.05^c^	4	2.5	ND	(6.36±0.71)x10^5^
**P10**	0.04^c^	3	2	ND	(5.46±0.66)x10^5^
**P11**	0.03^c^	3	2.5	ND	(1.55±0.35)x10^5^
**P12**	0.01^c^	4	2.5	ND	(3.82±0.45)x10^5^

^a^ T1 corresponds to the first transfection of BAC20-UL17mRFP in ESCDL-1.

^b^ Split ratio: area of ESCDL-1 cells (cm^2^) infected with a portion of the inoculum corresponding to 1 cm^2^ of the infected cell-culture flask.

^**c**^ multiplicity of infection in PFU/cell

**Fig 4:**
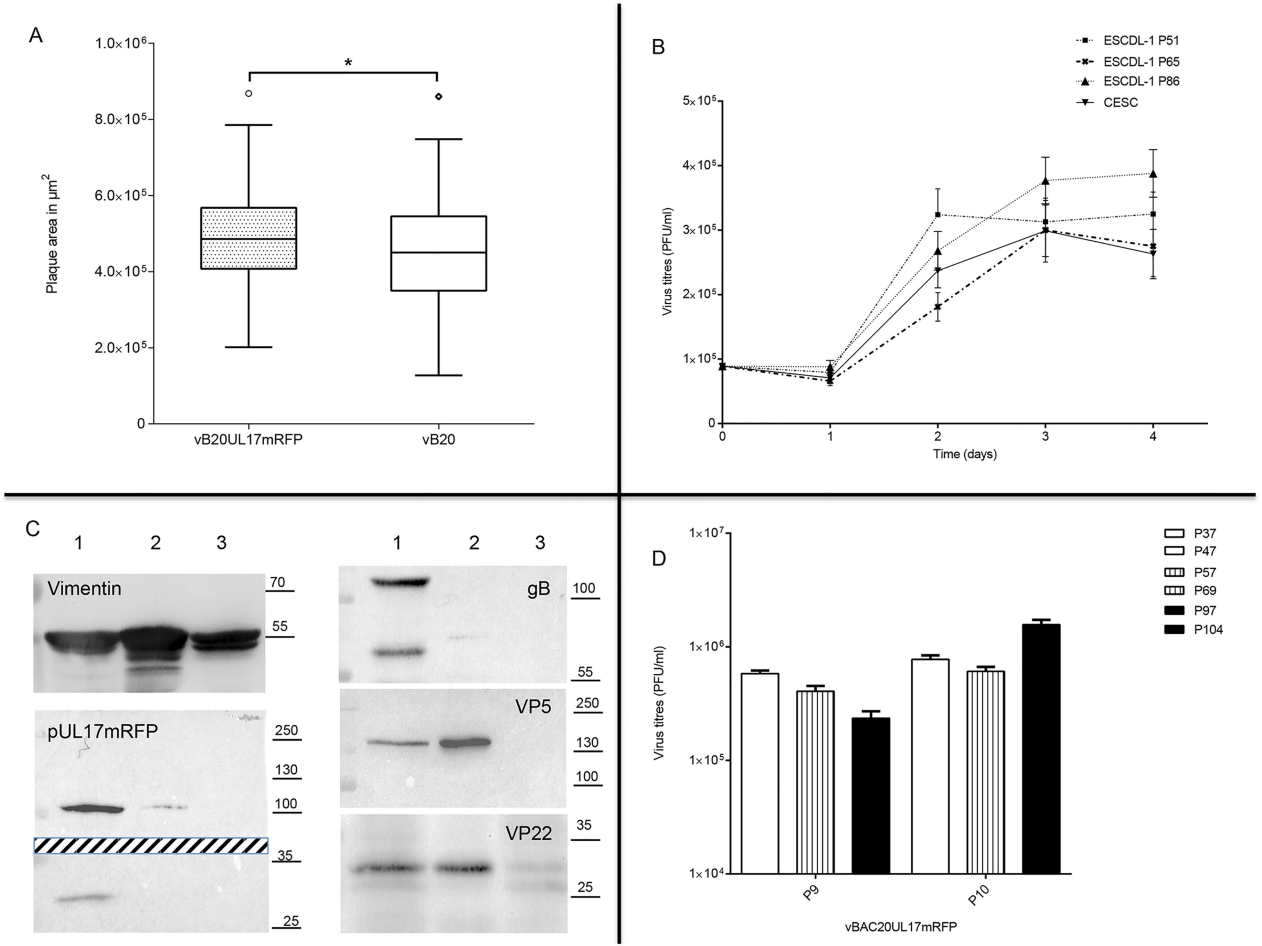
Replication of vBAC20UL17mRFP on ESCDL-1. (A) vBAC20UL17mRFP disseminates as efficiently as its parental virus vBAC20 on ESCDL-1. Viral plaques were counterstained with a mixture of monoclonal antibodies as described and areas were determined from a total of 180 plaques per virus in 2 experiments (Tukey box and whisker plots– Mann Whitney test—p = 0.015). (B) Kinetics of viral replication on ESCDL-1 at P41. ESCDL-1 cells were infected at an m.o.i of 0.02 and cell-associated virus was quantified from day 1 to day 4 on ESCDL-1 at intermediate or high passages, or on primary CESC (error bars represent 2 standard deviations). (C) Detection of viral proteins in cell extracts. Infected (lanes 1 & 2) or non-infected (lane 3) cell extracts were subjected to PAGE and blotting. Total cell-extracts were loaded in lane 1 and 3, while NP-40 insoluble cell extract was loaded in lane 2. Vimentin detection illustrates the relative amount of protein loaded. Protein pUL17-mRFP was detected by using a monoclonal anti-RFP; the upper part of the gel shows the detection of the fusion protein at the predicted apparent molecular mass (~110 kDa) while the lower part of the gel shows the presence of mRFP possibly cleaved from the fusion protein. (D) Comparison of vBAC20UL17mRFP production at passages 9 and 10 on low (P37-P47, white bars), intermediate (P57-P69, vertical bars) and high (P97-P104, black bars). The limited decline of virus production on intermediate / high passage cells at P9 was not confirmed at P10 (error bars represent 2 standard deviations).

We verified that former results on the comparison between vBAC20 and vBAC20UL17mRFP obtained on primary CESCs could be reproduced on ESCDL-1 by comparing the dissemination of the parental and recombinant viruses. Analysis of the results of plaque area measurements of vBAC20UL17mRFP (T1P4) and vBAC20 (T1P2) revealed a minor difference in plaque size, as the mRFP virus showed slightly larger plaques than the parental virus (Fig 4A). Kinetics analyses performed with passage 4 of vBAC20UL17mRFP at an initial M.O.I of 0.022 PFU/cell showed a progressive increase in viral titres (Fig 4B) paralleled by an increase in viral copy number (data not shown). Viral structural proteins VP5 (pUL19), VP22 (pUL49), gB (pUL27) and pUL17 fused to mRFP were readily detected in lysates from infected cells (Fig 4C). To further establish the potential of ESCDL-1 as a cell substrate for MDV replication, we compared ESCDL-1 to CESC on the basis of their respective virus production. For that purpose, we infected both cells in parallel with the same inoculum (vBAC20UL17mRFP at passage 4 –T1P4—at a MOI of 0.05), titrated the resulting viral progeny (vB20UL17mRFP—T1P5), which, in turn, was used to infect the matching cells (MOI of 0.002) from which the viral progeny was titrated (vB20UL17mRFP—T1P6). Viral inocula were collected in the same conditions for both cells and passages and virus titres were estimated on both cell types. Again, we observed no statistically significant differences relative to the cell type used for viral titration. Virus titres were almost identical for T1P5 virus production (1.89±0.15 x 10^5^ PFU/ml on primary CESCs vs 2.2±0.22 x 10^5^ PFU/ml on ESCDL-1) while infectious titres from T1P6 virus production on CESC were two-fold lower compared to those obtained for T1P6 production on ESCDL-1 (9.3±1.1 x 10^4^ vs 1.98±0.16 x 10^5^ PFU/ml, respectively). To evaluate whether the susceptibility of ESCDL-1 could vary over time, we passaged vBAC20UL17mRFP twice on ESCDL-1 at low (<50), intermediate (51<P<90) or high (>95) passages. Initial virus production was performed with T1P8 virus on ESCDL-1 at passages 37, 57 and 97 (MOI of 0.02), yielding T1P9 virus, which in turn was used to produce T1P10 virus on ESCDL-1 at passages 47, 69 and 104 (MOI of 0.01 to 0.02). The resulting viral progenies were titrated and no deleterious effect regarding the viral production on cells at high vs. low passages could be identified, over two serial passages (Fig 4D).

The permissiveness of ESCDL-1 for the replication of GaHV-2 was also assayed by using either vBACRB-1BUL17mRFP, the counterpart of vBAC20UL17mRFP in the RB-1B context, or vUL47-mRFP [50]. After transfection of either BAC, viral plaques were observed by day 5 or 6 post transfection. The resulting vBACRB-1BUL17mRFP or vUL47-mRFP were serially passaged twice and titrated in plaque assay. For vBACRB-1BUL17mRFP titres at passage 2 on ESCDL-1 reached 3.35±0.42 × 10^5^ PFU/ml and the titre of vUL47-mRFP at the same passage was 1.2±0.23 × 10^5^ PFU/ml.

The replication of HVT (MeHV-1) was also assessed on ESCDL-1 by using a first passage of FC126 virus on CESC as an inoculum, at a split ratio of 1/23. A second passage was performed on ESCDL-1 (at a ratio of 1/7) and titrated. The titres progressed from 3.5 (± 0.4) × 10^4^ in the inoculum to 1.61 (± 0.14) × 10^5^ for passage 2 on primary CESCs and 9.7 (± 0.62) × 10^4^ in ESCDL-1 to reach 1.73 (± 0.17) × 10^5^ for passage 3 (corresponding to the second passage in ESCDL-1).

The susceptibility of ESCDL-1 cells for GaHV-1 (ILTV) was also tested, in comparison with the LMH cells, and ESCDL-1 did not support ILTV replication (data not shown).

### Morphogenesis of cell-adapted GaHV-2 in ESCDL-1 is indicative of an efficient replication

**Fig 5:**
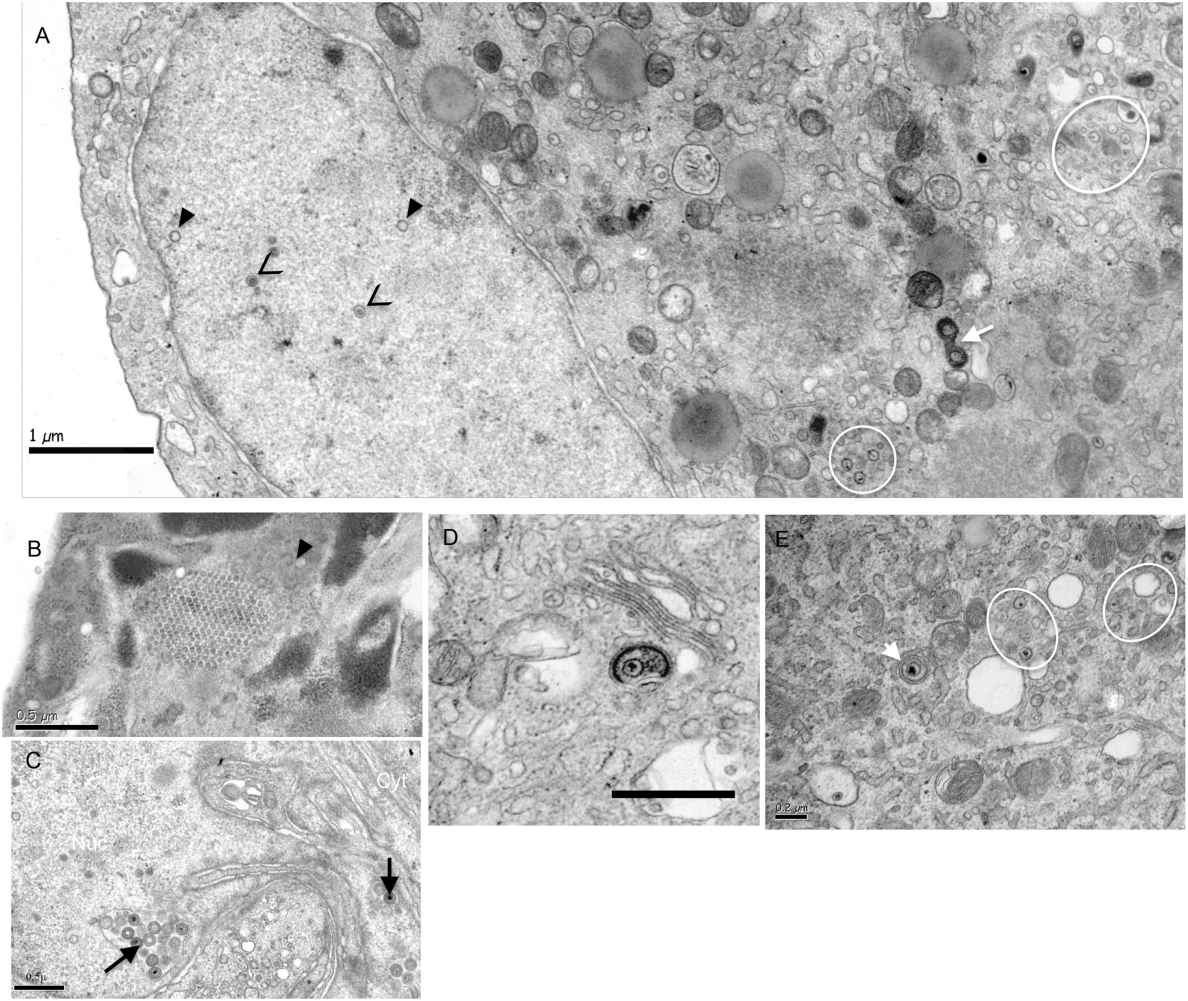
TEM analysis of vBAC20 morphogenesis in ESCDL-1. (A) Overview of an infected cell with intranuclear A (black triangle) and B (black arrowheads) capsids and intracytoplasmic C capsids (white ellipses). The white arrow points to an image of capsid tegumentation in the cytoplasm. (B) Intranuclear accumulation of small particles (SP– 30 to 35 nm in diameter) arranged in a pseudo-crystalline structure in the vicinity of A capsids (black triangle). (C) Accumulation of primary enveloped virions in distended cisternae of the nuclear membrane (black arrows point to enveloped C capsids). (D) C capsid undergoing secondary envelopment: electron dense material, possibly of tegument origin, surrounding the capsid is surrounded by a membrane in close vicinity to the Golgi (bar represents 0.2 μm). (E) Multiple intracytoplasmic C capsids (white ellipses) close to an enveloped cytoplasmic particle (white arrowhead).

A study of the morphogenesis on ESCDL-1 of cell-adapted GaHV-2 was performed by using vB20 at passage 2 after transfection of the corresponding BAC on ESCDL-1. Infected cell monolayers were harvested without enzymatic dissociation to preserve the integrity of cell connections and surface and collect cells at all stages of infection. Compared to the mock-infected cells (S5 Fig), infected ESCDL-1 were enlarged and rounded, often showing vacuoles containing viral material, and the nuclei displayed marginated chromatin together with nuclear membranes with a distended lumen (Fig 5A & 5B and S5 Fig). All stages of viral assembly previously described for GaHV-2 in primary cells [9] could be recorded and, as described in other cells, no extracellular enveloped viruses were observed. A total of 1084 particles (naked, tegumented and enveloped capsids) were scored in 29 infected cells, in which only 0.37% were enveloped intracytoplasmic particles (Table 2 and Fig 5D & 5E). Intranuclear A/B and C capsids were occasionally seen in the vicinity of small particle (SP—around 33 nm) aggregates (Fig 5B). Primary enveloped virions (PEV) were observed in distended cisternae of the nuclear envelope (Fig 5C) and accumulations of C capsids were often seen in the cytoplasm (Fig 5A). Images reminiscent of tegumentation were also noticed with electron dense material encircling or in close association with C capsids in the cytoplasm of infected cells (Fig 5A & 5D) or in vacuoles (S5B Fig).

**Table 2:** Number and percentage of capsids in the different cell compartments.

	Intranuclear capsids			Perinuclear capsids and primary enveloped virions (intraluminal)	Intracytoplasmic naked capsids		Intracytoplasmic tegumented (wrapping) capsids	Intracytoplasmic enveloped virions
Total / Percentage per compartment	656 / 60.52%			54 / 4.98%	356 / 32.84%		14 / 1.29%	4 / 0.37%
A/B/C capsid	A	B	C		A/B	C		
	123–11.35%	243–22.42%	290–26.75%		100–9.23%	256–23.62%		

### Generation of reporter and trans-complementing cell-lines in ESCDL-1

**Fig 6:**
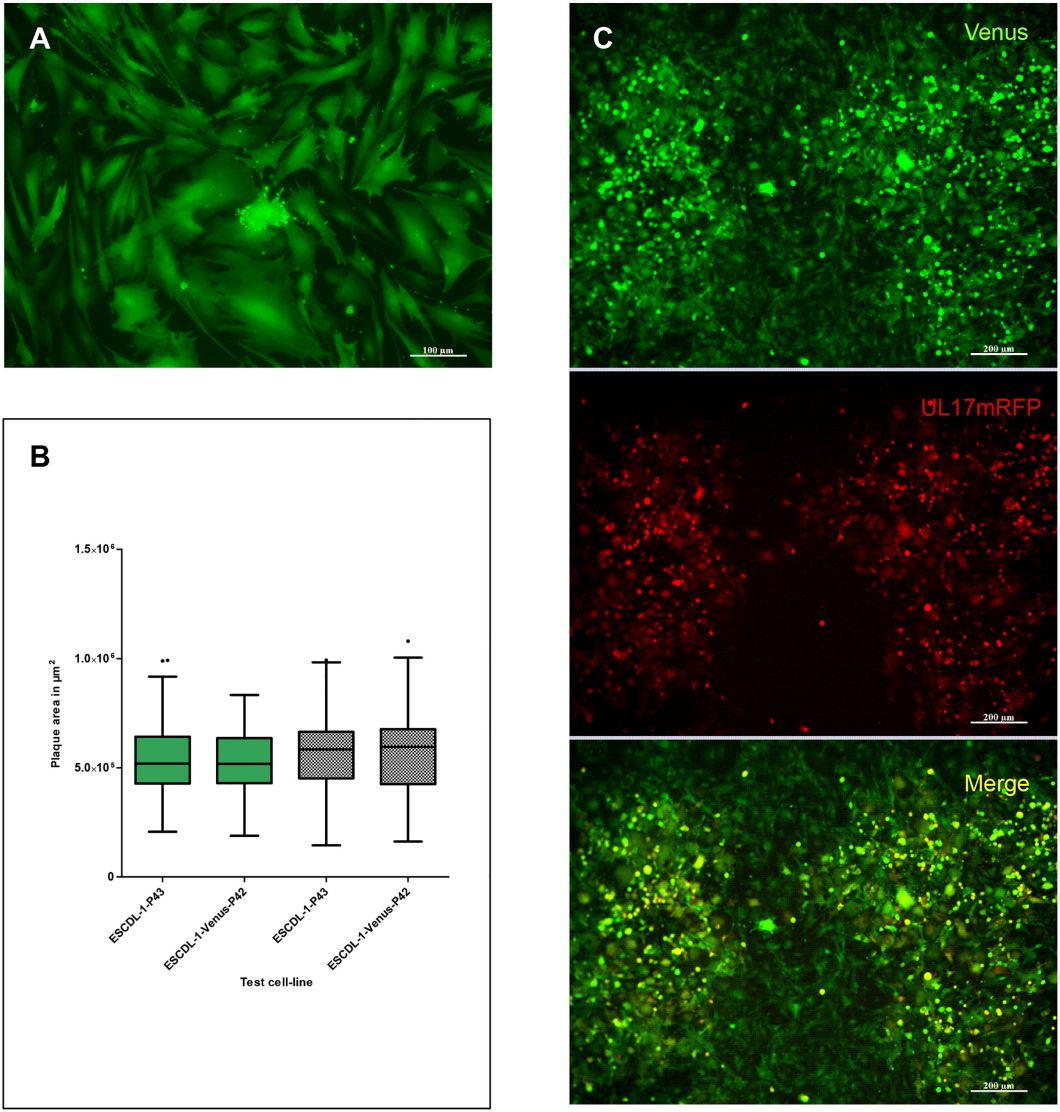
ESCDL-1 stably expressing the YGFP Venus support GaHV-2 replication. (A) Venus expression by ESCDL-1-Venus at passage 8 post selection initiation (scale bar indicates 100 μm). Venus YGFP is equally distributed in the nucleus and cytoplasm. (B) Comparison of plaque areas of vBAC20UL17mRFP between the parental cell line and the ESCDL-1-Venus/Clone7. The plaque areas of virus produced on ESCDL-1-Venus/Clone7 (green Tuckey box and whiskers plots) and on ESCDL-1 (shaded Tuckey box and whiskers plot) were measured for the parental and the Venus expressing cell-lines at matching passages (2 independent experiments). Differences existing between the cells or viruses were non significant (Mann-Whitney test with P values over 0.5). (C) vBACRB1BUL17mRFP plaques on ESCDL-1-Venus/Clone7: note the strong up-regulation of Venus expression in infected cells (scale bar indicates 200 μm).

The absence of a sustainable cell-line that would support Mardivirus replication has hampered the development of studies addressing the role of viral or cellular genes involved in viral morphogenesis and permissiveness. To further establish the usefulness of ESCDL-1, we first monitored the condition in which a stable clone expressing a transgene could be derived and whether the cloning process affected the permissiveness to GaHV-2. On the basis of its improved expression in eukaryotic cells, we chose to use the Venus variant of the yellow fluorescent protein (YFP) as a transgene [51]. From the ESCDL-1 transfected and directly exposed to hygromycin we observed the development of individual clones by day 12 post transfection (Fig 6) and stable cell clones expressing Venus YFP could be amplified and frozen in a period of 40 to 50 days. Cell populations expressing Venus YFP were also derived from the transfected cells exposed to hygromycin after one passage, in a period of time corresponding to 5 serial passages from the beginning of the selection (P22 > P27). Transgene expression could be easily visualized by fluorescence microscopy (Fig 6A) and western blot (data not shown). The permissiveness of one clone (ESCDL-1-Venus/Clone7 –EV7) was assessed in comparison with ESCDL-1 of matching passage. Virus vRB-1BUL17mRFP at passage 1 after transfection in ESCDL-1 was serially passaged twice on ESCDL-1 and on EV7 and titrated on ESCDL-1, EV7 and CESC (S3 Table). Viral titres were in the same range for both productions (10^5^ PFU/ ml) although approximately two-fold lower for the virus produced in EV7 (S3 Table). In the same experiment, plaque areas were measured for both virus productions and no statistically significant differences were found when comparing the viral dissemination on either the parental cells or the EV7 at matching passage numbers (Fig 6B). Interestingly the expression of Venus appeared to be enhanced by GaHV-2 infection (Fig 6C).

**Fig 7:**
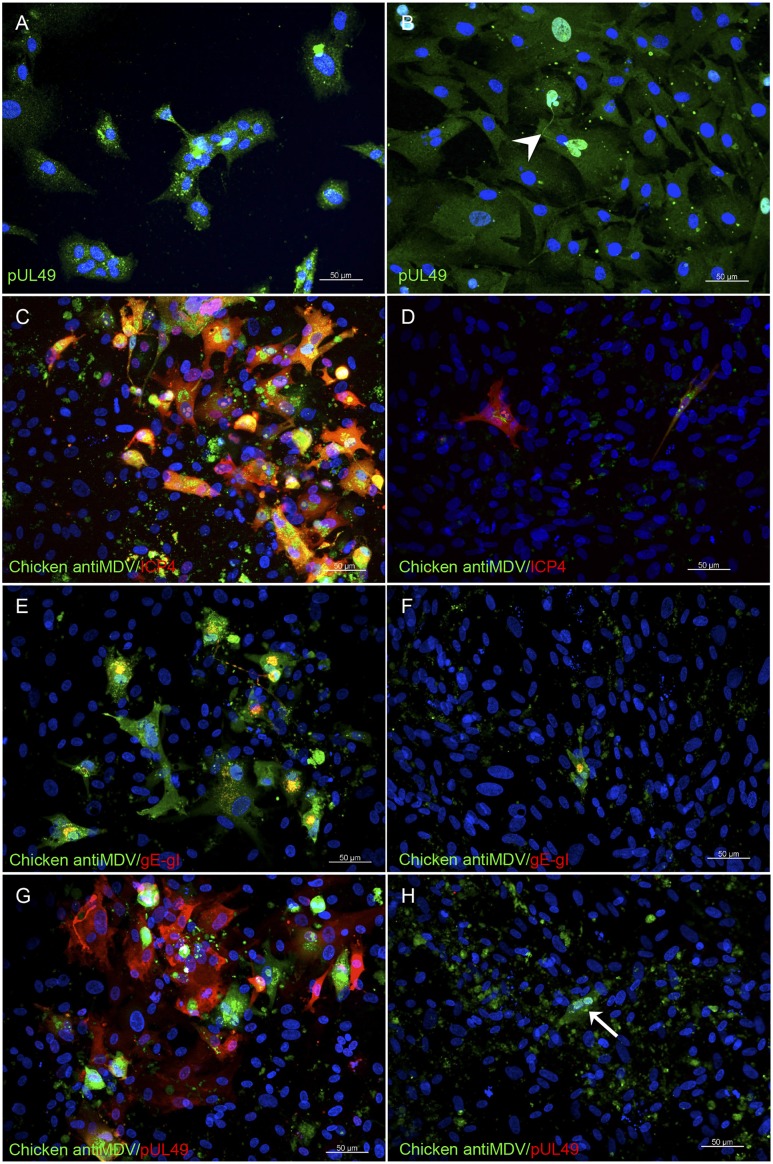
Constitutive expression of pUL49 (VP22) in ESCDL-1 complements the deletion of UL49 in GaHV2 genome. (A & B) Constitutive expression of pUL49 (VP22) in uncloned cell populations (A) and in ESCDL-1-UL49/clone 2 (B). VP22 staining by anti pUL49 Mabs and an Alexa Fluor^®^ 488 goat anti-mouse detects filamentous material between 2 strongly positive nuclei that appear to be still bound after cell division (white arrowhead). (C to H) Complementation of replication for BAC20ΔUL49 on ESCDL-1-UL49/clone 7 (C, E, G) and absence of viral dissemination in non-complementing ESCDL-1 (D, F, H). Viral replication was detected using a chicken hyper immune serum revealed by an Alexa Fluor^®^ 488 goat anti-chicken conjugate together with an anti-ICP4 Mab (C, D), a mixture of anti-gI and -gE Mabs (E, F), or a mixture of anti-pUL49 (VP22) Mabs (G, H) all revealed by an Alexa Fluor^®^ 594 goat anti-mouse conjugate. The restoration of pUL49 (VP22) expression is associated with the viral replication (G). Early-late (ICP4) and late (gE-gI) antigens are detected in isolated ESCDL-1 cells (D & F) and in panel H the arrow points to an isolated cell in which vBAC20ΔUL49 undergoes an aborted replication cycle as revealed by the polyclonal anti-MDV serum without detection of VP22. Scale bar represents 50μm.

Having established the proof of concept on the use of transgenic ESCDL-1 as a cell substrate for GaHV-2, we addressed the question of generating trans-complementing ESCDL-1 clones. We examined the trans-complementation of 2 GaHV-2 mutants in which the essential genes UL49 and UL37 had been invalidated [30]. Trans-complementing lines ESCDL-1-UL49 was generated by transfecting pCDNA3-UL49 [23] in ESCDL-1 at passage 32 and selection with G418. It is noteworthy that the selection procedure lasted 3 months, presumably due to the cell-toxicity of VP22 (pUL49). Expression of pUL49 could be monitored in both uncloned and cloned cell populations (Fig 7A & 7B). When BAC20ΔUL49 was transfected in complementing cells, viral plaques were detected (Fig 7C–7E–7G) and infectious virus could be retrieved from transfected ESCDL-1-UL49 while the transfection of the same BAC construct in ESCDL-1 yielded no viral plaques but only isolated cells stained for virus lytic cycle protein (Fig 7D–7F–7H).

**Fig 8:**
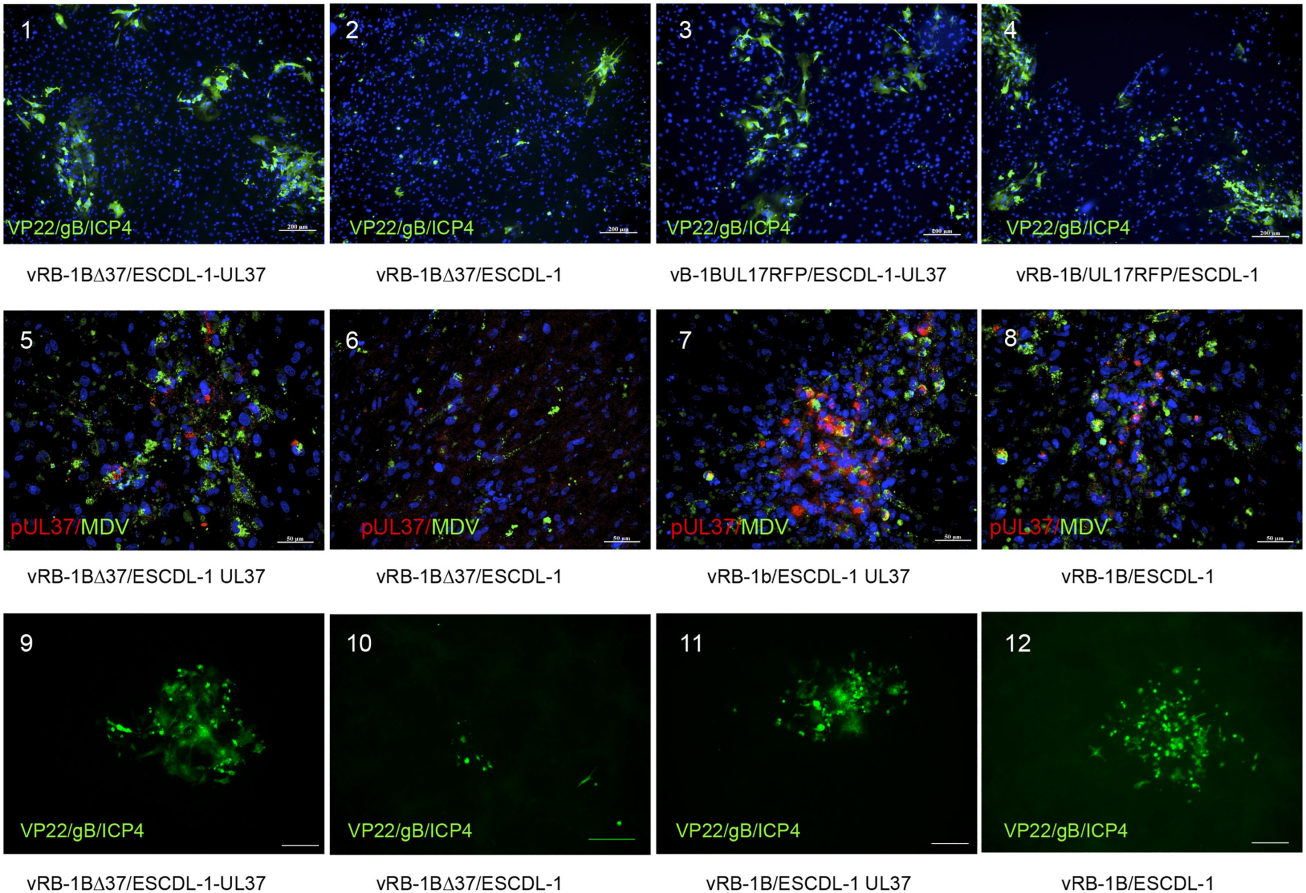
Expression of pUL37 in ESCDL-1 complements the deletion of UL37 ORF in BACRB-1B. Upper panel (1–4): Transfection of BACRB-1BΔ37 yields viral plaques on complementing cells: viral plaques were detected in ESCDL-1-UL37 by staining with Mabs B17 (anti-VP22), K11 (anti-gB) and E21 (anti-ICP4) and an Alexa Fluor^®^ 488 GAM conjugate (1). In ESCDL-1, BACRB-1BΔ37 did not yield a virus that could disseminate and only isolated positive cells could be seen (2). As a control, BACRB-1BUL17mRFP was transfected in either complementing or non-complementing parental cells, producing viral plaques on both (3 & 4). Scale bar = 200 μm. Middle Panel (5 to 8): vBACRB-1BΔ37 can be serially passaged in complementing cells and virus multiplication induces pUL37 expression in the ESCDL-1-UL37: BACRB-1BΔ37 (5,6) or BAC RB-1B (7,8) were transfected either in ESCDL-1-UL37 complementing cells or in ESCDL-1 and passaged once in the same cells. The development of viral infection by passage 2 of the vBACRB-1BΔ37 virus is seen in complementing cells (green fluorescence in 5) and coincides with the expression of pUL37 in infected cells (red fluorescence in 5); in non-complementing cells the same virus passage does not replicate (6). The parental virus (vBACRB-1B) transfected and passaged in the same conditions replicated equally well on ESCDL-1 and on ESCDL-1-UL37 (7 & 8). Scale bar = 50μm. Lower Panel (9 to 12): vBACRB-1BΔ37 may be passaged at least 3 times in complementing cells and does not revert to a replicating virus when plated on non-complementing cells. The 3rd passage of vBACRB-1BΔ37 yielded typical viral plaques in complementing cells (9) whereas the same virus did not form plaques in ESCDL-1 (10). Again vRB-1B at the same passage developed equally well in both cells (staining as in the upper panel, except for HOECHST 33342). Scale bar = 200 μm.

**Fig 9:**
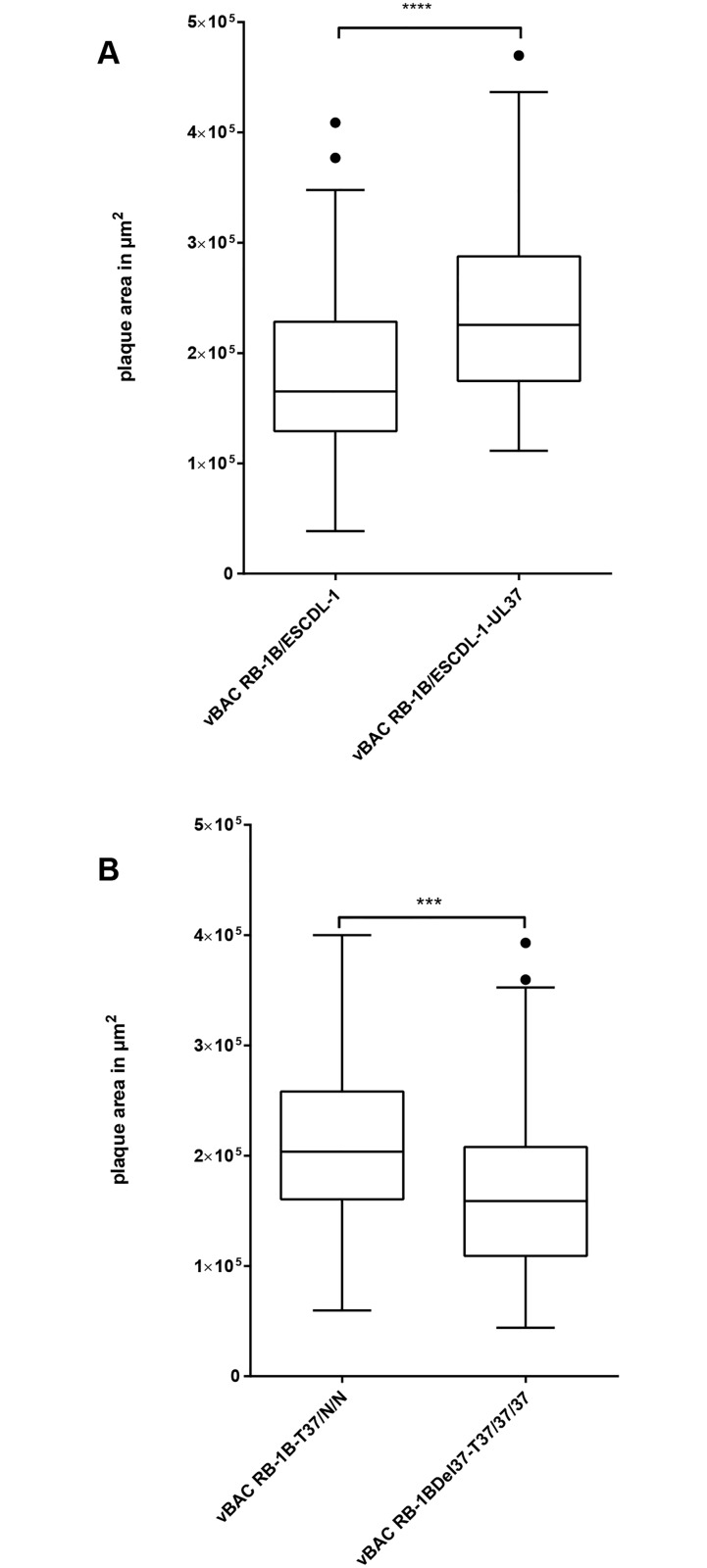
vBACRB-1BΔ37 and vBACRB-1B dissemination in complementing and parental ESCDL-1. (A) Comparison of dissemination of vBACRB-1B on ESCDL-1 or ESCDL-1-UL37: plaque areas were significantly larger on ESCDL-1-UL37 (Tukey box and whiskers plot—P< 0.0001, Mann Whitney test). (B) Comparison of plaque areas of parental and Δ37 viruses at their 3^rd^ passage (on complementing cells only for the Δ37 virus). Both viruses were plated on ESCDL-1-UL37 and plaque areas were measured at day 4 after plating. Statistically significant differences are indicated with an asterisk (Tukey box and whiskers plot—P = 0.0004, Mann Whitney test).

The deletion of UL37 ORF in MDV was reported to be highly deleterious, yielding non replicating mutants in the BAC20 backbone [52] and the effect was similar in the BACRB-1B backbone. We ascertained that the UL37 deletion resulted in the replication defect as the deletion mutant was rescued by co-transfecting BACRB-1BΔUL37 with a complementing PCR product encompassing the whole UL37 ORF (data not shown). A cell population of ESCDL-1 stably transfected with pCDNA3 UL37, ESCDL-1-UL37, was obtained in the conditions described above, within 30 days from the initial transfection to the freezing of cell stocks. The expression of pUL37 was found to be elusive (data not shown) and the cells were tested for complementation of BACRB-1BΔ37 in comparison with ESCDL-1 at matching passage number. Upon transfection of BACRB-1BΔ37 visible plaques appeared 6 days post transfection in complementing cells; in contrast no CPE could be seen in non-complementing cells. Control transfection of a BACRB-1BUL17mRFP yielded plaques after 4 days in both cell lines. Replication was confirmed in the complementing ESCDL-1 UL37 by IIF of early/late and late phase antigens, while only isolated cells expressing late phase antigen could be seen in non-complementing ESCDL-1 (Fig 8 panels 1 to 4). Expression of pUL37 was detected in the context of infected cells in trans-complementing ESCDL-1 (Fig 8 –panel 5). We further explored the possibility of serially passaging the deleted virus on the complementing cells, in comparison with the parental virus. The development of viral plaques was monitored at passage 2 and 3 (Fig 8) and plaque sizes were measured (in complementing cells only for the Δ37 virus). When vBACRB-1BΔ37 at the 3^rd^ passage in complementing ESCDL-1 UL37 was plated on complementing cells, viral plaques were observed whereas plating the same virus on non-complementing ESCDL-1 yielded at best “phantom plaques” apparently made of dying cells from the inoculum (Fig 8 panel 10). The cell-to-cell dissemination of the mutated virus was estimated from the comparison between plaque areas of vRB-1BΔ37 at passage 3 and vRB-1B (Fig 9). We first measured the areas of plaques from the parental vBACRB-1B either on ESCDL-1 or ESCDL-1 UL37 (Fig 9A) and found that the trans-complementing cell-line apparently supported an improved cell-to-cell dissemination. The vBACRB-1BΔ37 virus induced viral plaques that were smaller than those of the parental virus (Fig 9B) in the trans-complementing ESCDL-1 UL37. It is noteworthy that the average diameter of vBACRB-1BΔ37 plaques in the complementing ESCDL-1 was in the range of that of vBACRB-1B on ESCDL-1 (Fig 9). From this set of experiments, we concluded that ESCDL-1 are the first described cell-line from which several trans-complementing cell populations can be derived, enabling further analysis of viral and cellular protein involvement on GaHV-2 replication and dissemination.

## Discussion

In this study, we show that the acquisition of susceptibility to GaHV-2/Mardiviruses is dependent on the differentiation of cells derived from cES cells. Indeed, when maintained as rapidly growing ES cells, the cES cells were refractory to GaHV-2 replication; in contrast, once induced to differentiate, these cells became permissive to infection. This absence of susceptibility of ES cells has been reported for herpesviruses such as VZV in human ES cells [21] or mouse cytomegalovirus in mouse ES cells [53], and in both cases, permissiveness was shown to depend on differentiation. We developed a strategy based on the use of small molecule induced differentiation and used viral permissiveness as the sole criterion to evaluate the effects of the molecules. In the format of a direct test on cES cells, we established that HMBA and, to a lesser degree, ATRA induced a differentiation resulting in the acquisition of susceptibility to GaHV-2 for cES derived cells to GaHV-2. Retinoic acid derivatives are potent regulators of cell differentiation, inducing ES cells differentiation toward the neurectodermal lineage [54, 55]. The prototypical hybrid polar compound HMBA has been extensively studied for its differentiating activity on leukemic and solid tumor cell lines [56, 57] and shown to compensate the effects of the deletion of VP16 activation domain on HSV-1 mutant replication [58]. It is noteworthy that both drugs are known to induce PBX/HOX transactivation factors that play a role in organ development [59] and modulate late antigen expression in VZV infected cells by binding to promoter regions upstream VZV ORF4 and ORF14 (gC) [45].

Although both chemicals induced the susceptibility to GaHV-2 in differentiated cES cells, we rapidly came to the conclusion that direct differentiation of cES cells was not compatible with current virology studies and we developed a protocol for differentiation that could lead to the establishment of a cell line supporting Mardivirus replication. From cES cells exposed to the differentiating stimuli described in our protocol, we derived the ESCDL-1 that were further characterized after initially testing permissive for GaHV-2 replication. The expression of mesenchyme lineage markers by ESCDL-1, in particular with no detectable expression of cytokeratins, high expression of VIM, THY1, NGF, ENDOG and PDGFRB genes and high expression of the FOXC2 specific transcription factor, is in good agreement with the known effect of HMBA as a strong inducer of markers of the mesodermal lineage in human ES cells [60]. We also report on the differential expression of numerous integrins including ITGB5, which was shown to play a major role in Epithelial-Mesenchymal-Transition (EMT) [61] and of specific forkhead transcription factors, members of the CEBP and FOX families, all indicating a strong connexion with the mesenchymal phenotype for ESCDL-1. The further molecular characterization of the ESCDL-1 cells suggests a cell type able to secrete and produce numerous growth factors from the FGF family (FGF7, FGF16), the TGF-beta superfamily (GDF6 (BMP13), TGFB1, TGFB3, GREM2), the IL6 family (IL6, LIF, IL15), and the WNT family (WNT9A, SFRP4). Markers such as ACVRL1/ALK1, EPHB1, AGTR2, SCARB1/CD36, TNFRSF14/CD270, TNFRSF5/CD40, THY1/CD90, THBD/CD141, among others, indicate a cell type able to be stimulated through specific membrane bound receptors. All together, on the basis of telomerase activity, robust multiplication, strong contact-inhibition of replication, and a stable phenotype, we suggest that ESCDL-1 may be best described as an immortalized non-transformed cell-line with mesenchymal features.

Study of permissiveness of ESCDL-1 encompassed testing the serial passage of pathogenic and non-pathogenic cell-adapted GaHV-2 and of MeHV-1 on ESCDL-1, in comparison with primary CESCs. In all tests run in parallel, ESCDL-1 compared equally or better to primary CESCs; however, it remains difficult to compare with previously published data as, especially for GaHV-2, split ratio and freezing methods often vary from one publication to another. In our studies though, the viral titres obtained in ESCDL-1 are in the range of those described for SogE-QM7 cells [14]. Also, as described earlier for the QM7 derived line [14], ESCDL-1 were fully susceptible to all GaHV-2 tested, which did induce a cytopathic effect at their first passage, unlike in JBJ-1 cells [15]. In addition to the evaluation of viral susceptibility, we show here evidence for a permissiveness that is unaffected by the number of serial passages, a feature of major interest for virology studies.

The morphogenesis study was conducted using vBAC20 at a low passage after its transfection in ESCDL-1 and exploiting a cell culture system that allowed the retrieval of cells without enzymatic dissociation. When comparing with previous data obtained in primary CEF with the same virus [62], we observed a slightly lower percentage of intranuclear capsids (60.5 vs 75%), and consequently, a higher percentage of both PEV in the nuclear membrane lumen (4 vs 1%) and intracytoplasmic naked capsids (32.8 vs 24%). The percentage of intracytoplasmic enveloped virions was similar to the one reported for vBAC20EGFPVP22 [9] and for vBACRB-1BUL47GFP [63]. The higher number of vBAC20 particle per cells in ESCDL-1 (37.3) compared to primary CEF (6.35) or to vBAC20EGFP VP22 in primary CESCs (10.1) may indicate a more efficient egress from the nucleus. Together with this efficient nuclear egress, we confirmed the persistence of the blockade of secondary envelopment described with vBAC20EGFPVP22. Comparison with HSV morphogenesis also enlightens this blockade as percentages are almost twice higher for intranuclear capsids (60.5 vs 34 to 40%) and three times higher for intracytoplasmic C capsids (32.8 vs 10%) in MDV infected cells compared to HSV [64, 65]. Interestingly, several images suggesting difficulties in the maturation of a tegument were recorded together with frequent accumulation of C capsids in the cytoplasm. This is also reminiscent of secondary envelopment blockade associated in HSV-1 with deletion of tegument or glycoprotein genes (for a review see [66]). Whether this blockade is relieved in FFE remains to be ascertained, but the differential expression of several tegument proteins [67] might be indicative of the progression of MDV toward a morphogenesis process yielding infectious cell-free particles. However the sole over-expression of UL48 in a BAC context [67] or the expression of UL48 in ESCDL-1 (data not shown) did not relieve the impairment in viral morphogenesis in cell culture.

Using a reporter gene, we show that ESCDL-1 are resilient cells, being able to endure transfection, cloning and to support the expression of foreign transgenes, without loosing their susceptibility to GaHV-2. The Venus expressing ESCDL-1 were used to build on the proof-of-concept of this major feature, but those cells might be of significant value *per se* in designing experiments aimed at deciphering the mechanism of cell-to-cell viral infection in the MDV model.

We have also established the feasibility of the complementation in trans in ESCDL-1 by using 2 tegument genes that were shown to be essential for MDV dissemination *in vitro*. The selection of cells complementing for UL49 has been described as difficult, due to intrinsic cell toxicity of VP22, leading to the utilization of either inducible promoters [68] or baculovirus-mediated UL49 expression [69]. We experienced a lengthening of the selection period for ESCDL-1 UL49, but eventually selected a trans-complementing cell-line. In our pioneering study showing that GaHV-2 UL49 gene was indispensable [30], we reported on a limited complementation in trans by UL49 expressing QM7, but we could not, at that time, establish the cause of this limitation, which could be due to the limited susceptibility of the QM7 for GaHV-2 or to the cell-toxicity of UL49 [35]. The comparison with ESCDL-1 now leads us to suggest that the initial limited susceptibility of QM7 was the major cause of inefficient complementation. It has indeed been reported that such limitation or absence of permissiveness to BoHV-4 could be overcome by expression of viral genes (IE2) in human rhabdomyosarcoma cell line RD4 [70], also suggesting that constitutive viral gene expression may increase cell susceptibility when the latter is intermediate or low, but have no effect on permissiveness in fully susceptible cells. We focussed on the complementation of another essential gene coding for a tegument protein, UL37, in the RB-1B backbone and showed that pUL37 expressing ESCDL-1 (ESCDL-1-UL37) complemented this deletion and supported at least 3 rounds of serial replication. The parental ESCDL-1 line failed to support the replication of the deleted virus and no spontaneous reversion was observed. However, the use of vRB-1BΔ37 to decipher the role of pUL37 in virus morphogenesis, as described for HSV-1 [71, 72], was impossible for GaHV-2, mainly due to the rather low viral titres obtained and to the absence of extracellular virion production. For both gene complementation strategies we chose to clone viral genes under the well-known P_CMV IE_ promoter for expression in ESCDL-1, as the endogenous promoters are not precisely defined. Initially we observed a strong transactivation of P_CMV IE_ promoter in Venus-expressing ESCDL-1 infected by GaHV-2 and we also observed an apparently beneficial transactivation of this promoter yielding an increase in viral transgene expression in infected trans-complementing cells. Transactivation of plasmid borne promoters by herpesviruses was reported long ago [73], and we considered it as beneficial in our trans-complementing strategy. Indeed viral transgenes are often silenced in cell clones, as reported for HSV-1 UL36 [74], and as observed in ESCDL-1-UL37. Trans-complementation has been reported for GaHV-2 [14, 28]; however, we claim that ESCDL-1 provides a better cell substrate with increased susceptibility compared to QM7, as illustrated by the comparison of the results obtained on QM7- or ESCDL-1-UL49.

Through this description of ESCDL-1 and from our previous results on cES cell-derived keratinocytes [20], we propose that cES cell lines may provide a reliable source of continuous cell-line derived by the use of differentiating protocols that may be elaborated from a rationale built on the knowledge of virus/cell interactions.

### Ethics statements

Primary chicken skin cells (CESCs) were prepared as described [23] from 12 day-old embryos of LD1 Brown Leghorn chicken line. This procedure was carried out in strict compliance with the French legislation for animal experiments which states that the use of embryos from oviparous species before the last third of their development (i.e. before day 14 for chicken embryos) is not submitted to regulation (Art. R.214-88).

## Supporting information

S1 Fig Monoclonal antibodies to pUL37 and BACRB-1BΔUL37 characterization. A) Monoclonal antibody AA8 stains Baculovirus-UL37 infected Sf9 cells; Nuclei were counterstained with Hoechst 33342 and an AlexaFluor488 goat anti-mouse conjugate was used to reveal the bound antibodies (bar represents 50μm). B) RFLP analysis of mutated BACRB-1B clones after *Bln*I digestion (B.a) and Southern blotting (B.b) of BACRB-1BΔUL37 clones 1 & 2 (1,2) compared to BACRB-1BΔUL47 clones (3, 4, 5) and pRB-1B BAC clone (6, 7). *Bln*I digestion of BACRB-1BΔ37 generates an additional fragment (A 1 and 2—arrowhead). As a control BACRB1BDel47 clones (lanes 3 to 5) and BACRB1B (lanes 6 and 7) were submitted to the same digestion. The KanR gene in BACRB1B-Del37 is associated with a 5560 bp fragment (B.b lanes 1 and 2) corresponding to *Bln*I fragment 85095–90660. In BACRB1B-Del47, a 31,5 kbp fragment contained the KanR cassette. The “SmartLadder” from Eurogentec was used as a size standard (M).
(TIF)

S2 Fig Replication characteristics of ESCDL-1. Telomerase activity (A), cell-cycle (B) and growth curve. A) Telomerase activity of CESC (1), DF1 (2), LMH (3), ESCDL-1 (4) and CLEC 213 (5) cells. B) Cell cycle analysis of ESCDL-1 and primary CESC at 24 and 48 h post plating. C) Growth curve of ESCDL-1 over 25 days.
(TIF)

S3 Fig Protein-protein network. The networks were obtained by using the STRING software with the first 150 most differentially expressed genes in ESCDL-1 compared with the initial cES cells as listed on S3 File.
(TIF)

S4 Fig Various expression profiles are observed for genes up or down-regulated by the HMBA treatment. The expression of different genes were validated by real time RT-PCR, including ECM2, CPM, DPT and FABP4 found down-regulated in CESC after HMBA treatment (A), OLFM3, IGFBP2, ID2 and THBS2, found up-regulated in CESC after HMBA treatment (B), NANOG, SCNN1A, LCN8 and ATP12A, found down-regulated in cES cells after HMBA treatment (C), KRT15, SCD5, WISP1 and THBS4 found up-regulated in cES cells after HMBA treatment (D), NAPEPLD, LGALS1, IRF1 and AK1 found down-regulated in both CESC and cES cells after HMBA treatment (E), MGAT3, HS6ST3 and TERG1L found up regulated in both CESC and cES cells after HMBA treatment (F and ARAP3, LOC421054, LOC422654 and CDKN2A found down-regulated in CESC, but up-regulated in cES cells after HMBA treatment (G). Expression was also analysed in CEF and DF1 fibroblasts, in LMH, and in ESCDL1 cells. Expression was taken to be 1 in cES as a reference, and two independent samples were run, each in triplicate. Error bars indicate SD.
(TIF)

S5 Fig TEM analysis of non-infected and infected ESCDL-1. A and A’) Overview of a non-infected cell. Due to the elongated shape of the cells only the nucleus region is presented to illustrate the characteristic morphology of these cells. A’ inlet shows a magnification of the boxed zone in A. B) Overview of a highly infected cell undergoing vacuolization. Intranuclear A/B/C capsids are present and a primary enveloped particle can be seen in the nuclear membrane lamina (magnification of the boxed area). Intracytoplasmic capsids are encircled in white in the boxed area. Numerous A and C capsids can be seen in the vacuole (magnification of the boxed area, black arrowheads) in which electron dense material reminiscent of viral tegument particle (L particles in HSV-1) is also present (black circle).
(TIF)

S1 File List of all differentially expressed genes with the GO term analysis. (XLSX)

S2 File Short list of the genes differentially expressed in both the CESC and cES cells following the HMBA treatment. (XLSX)

S3 File List of differentially expressed genes as revealed by RNA sequencing in primary fibroblasts (CEF), cES cells (cES1 and cES2) and ESCDL1 cells. (XLSX)

S1 Table List of oligonucleotides used in real time RT PCR analyses. (DOCX)

S2 Table Differential gene expression in HMBA treated vs non-treated cES cells compared to CESC. (DOCX)

S3 Table Viral replication on ESCDL-1-Venus (EV7) compared to the parental ESCDL-1. (DOCX)
