# Supplementary figures and images for: ESCDL-1, a new cell line derived from chicken embryonic stem cells, supports efficient replication of Mardiviruses
Source: PLoS One. 2017 Apr 13;12(4):e0175259. doi: 10.1371/journal.pone.0175259 (PMC5391029; doi:10.1371/journal.pone.0175259)

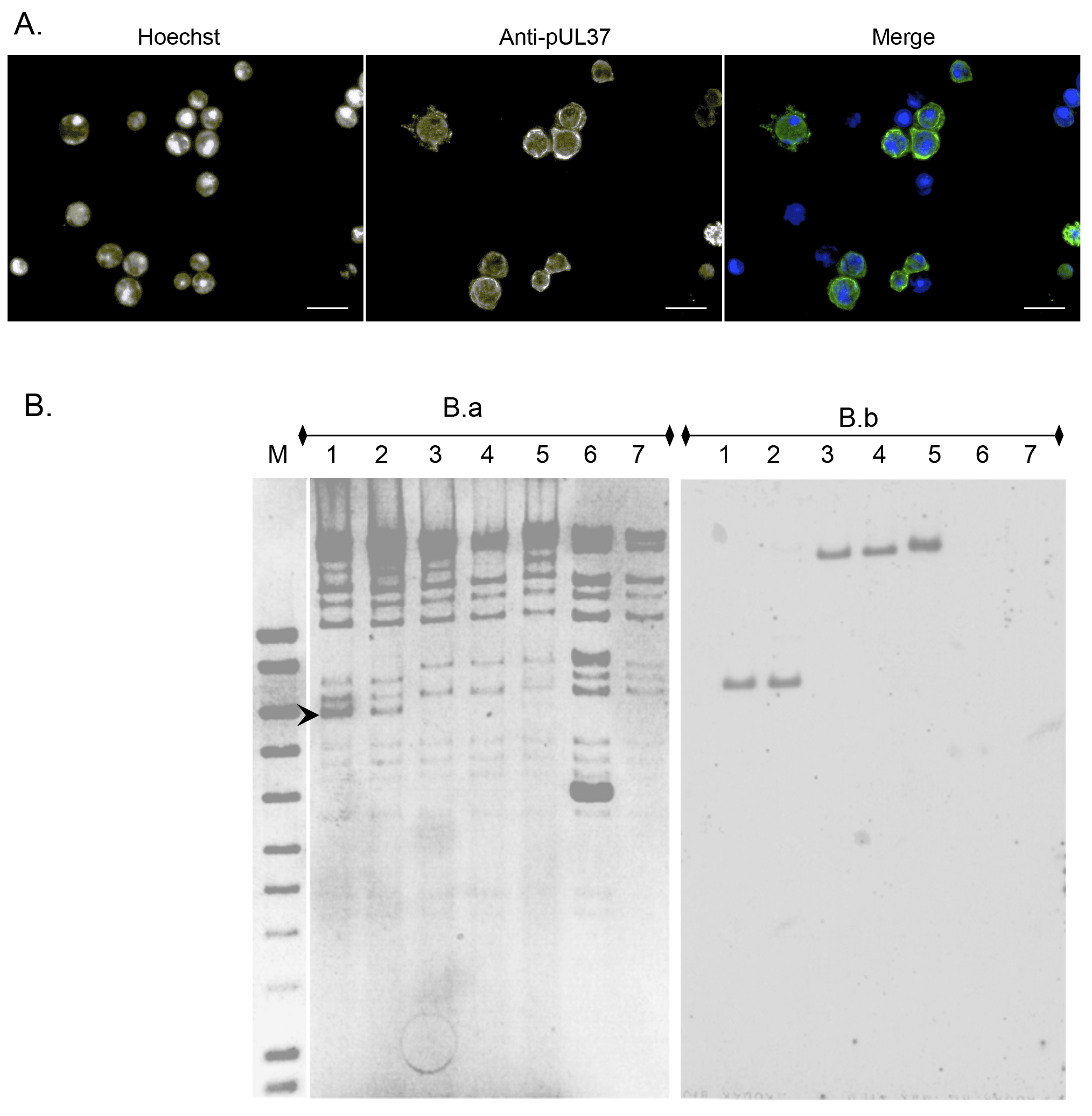

Supplement: S1 Fig — A) Monoclonal antibody AA8 stains Baculovirus-UL37 infected Sf9 cells; Nuclei were counterstained with Hoechst 33342 and an AlexaFluor488 goat anti-mouse conjugate was used to reveal the bound antibodies (bar represents 50μm). B) RFLP analysis of mutated BACRB-1B clones after BlnI digestion (B.a) and Southern blotting (B.b) of BACRB-1BΔUL37 clones 1 & 2 (1,2) compared to BACRB-1BΔUL47 clones (3, 4, 5) and pRB-1B BAC clone (6, 7). BlnI digestion of BACRB-1BΔ37 generates an additional fragment (A 1 and 2—arrowhead). As a control BACRB1BDel47 clones (lanes 3 to 5) and BACRB1B (lanes 6 and 7) were submitted to the same digestion. The KanR gene in BACRB1B-Del37 is associated with a 5560 bp fragment (B.b lanes 1 and 2) corresponding to BlnI fragment 85095–90660. In BACRB1B-Del47, a 31,5 kbp fragment contained the KanR cassette. The “SmartLadder” from Eurogentec was used as a size standard (M). (TIF) [file pone.0175259.s001.tif]

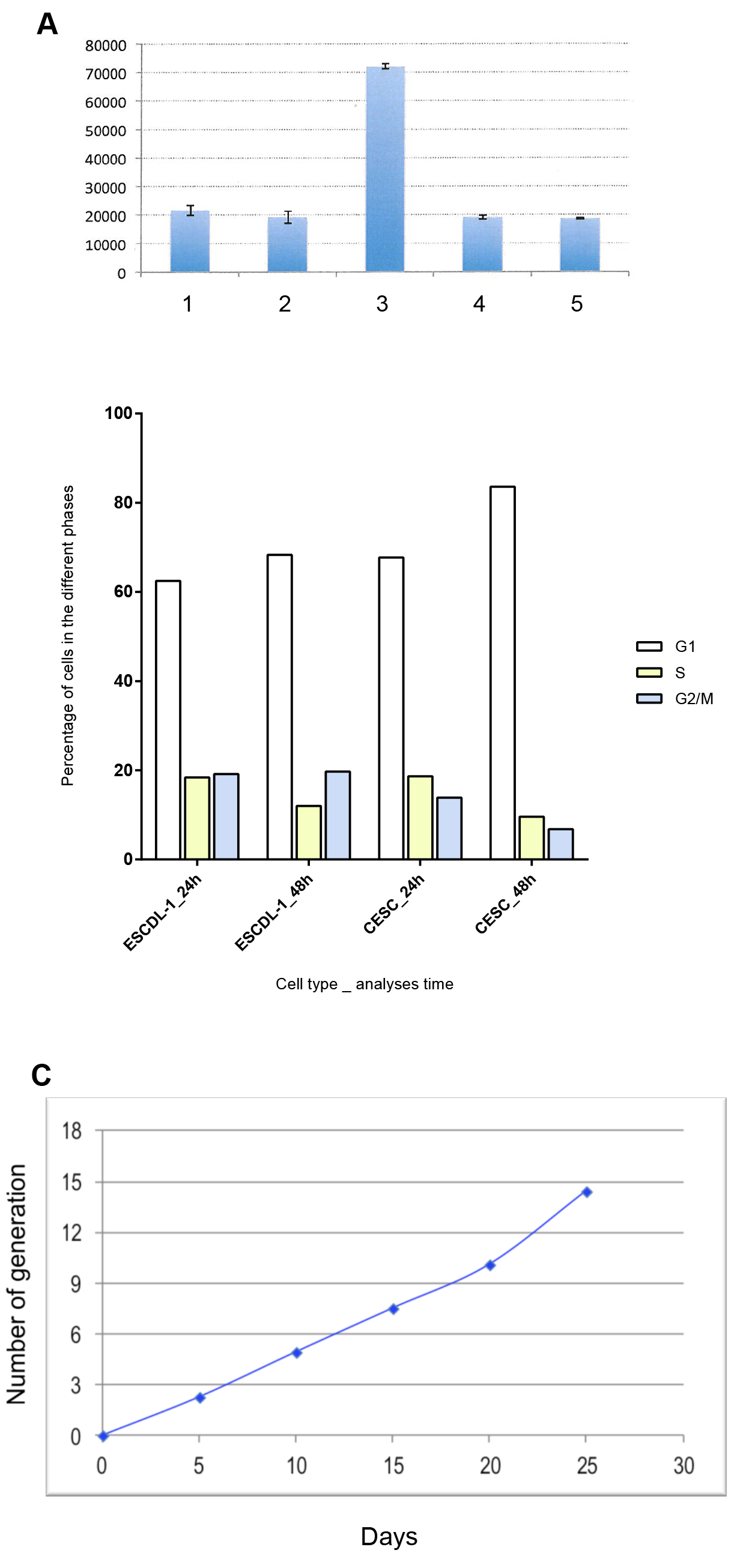

Supplement: S2 Fig — Telomerase activity (A), cell-cycle (B) and growth curve. A) Telomerase activity of CESC (1), DF1 (2), LMH (3), ESCDL-1 (4) and CLEC 213 (5) cells. B) Cell cycle analysis of ESCDL-1 and primary CESC at 24 and 48 h post plating. C) Growth curve of ESCDL-1 over 25 days. (TIF) [file pone.0175259.s002.tif]

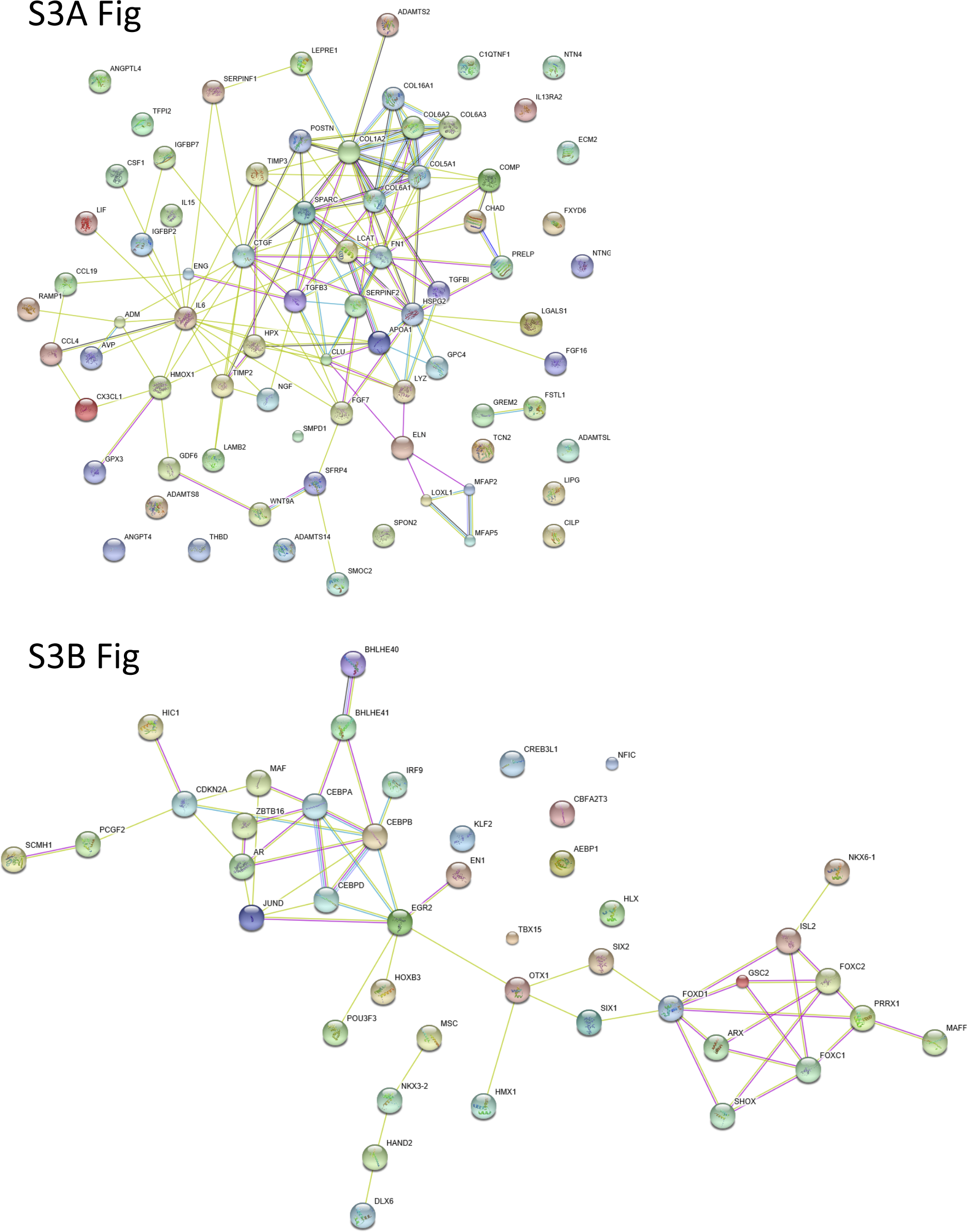

Supplement: S3 Fig — The networks were obtained by using the STRING software with the first 150 most differentially expressed genes in ESCDL-1 compared with the initial cES cells as listed on S3 File. (TIF) [file pone.0175259.s003.tif]

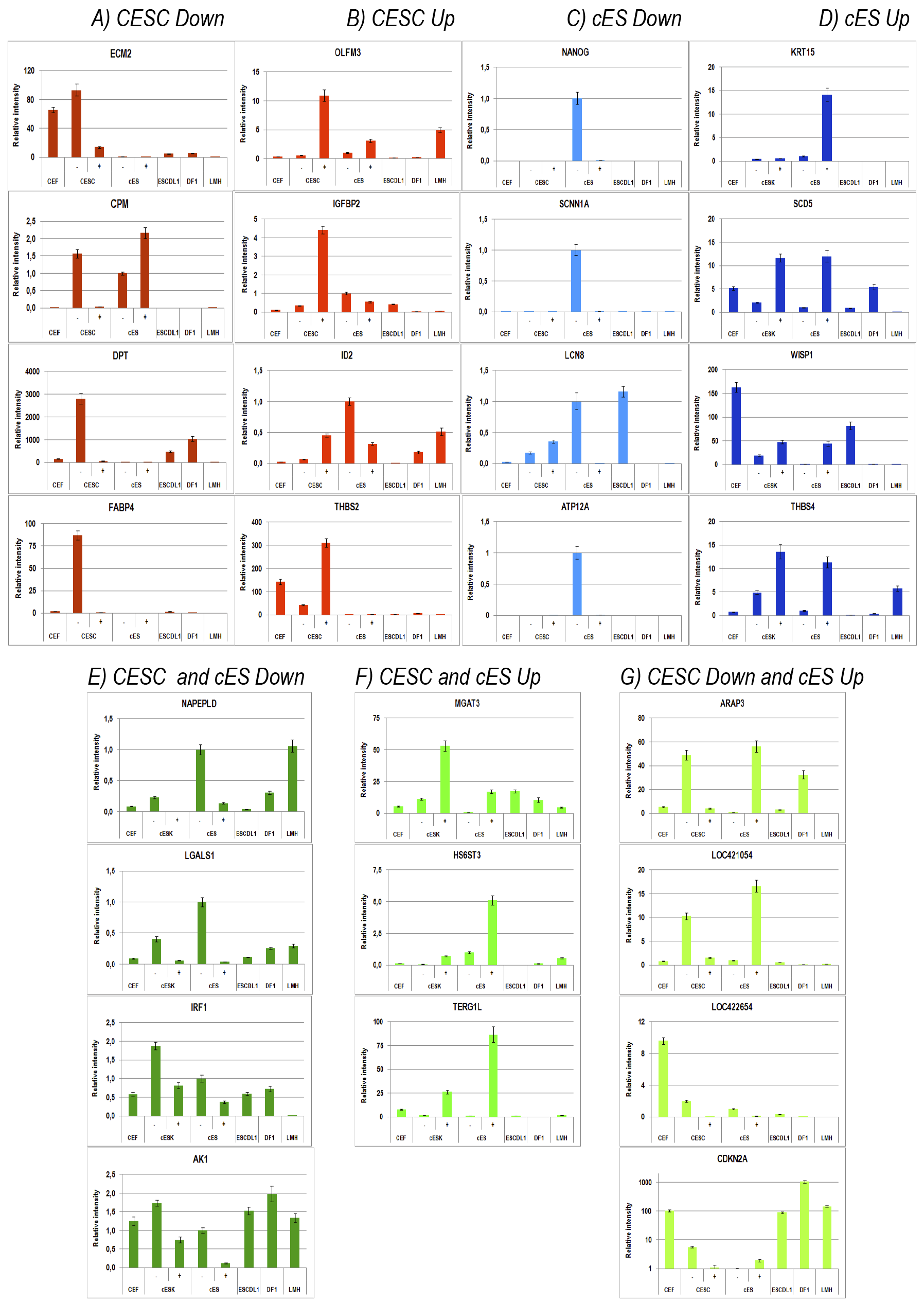

Supplement: S4 Fig — The expression of different genes were validated by real time RT-PCR, including ECM2, CPM, DPT and FABP4 found down-regulated in CESC after HMBA treatment (A), OLFM3, IGFBP2, ID2 and THBS2, found up-regulated in CESC after HMBA treatment (B), NANOG, SCNN1A, LCN8 and ATP12A, found down-regulated in cES cells after HMBA treatment (C), KRT15, SCD5, WISP1 and THBS4 found up-regulated in cES cells after HMBA treatment (D), NAPEPLD, LGALS1, IRF1 and AK1 found down-regulated in both CESC and cES cells after HMBA treatment (E), MGAT3, HS6ST3 and TERG1L found up regulated in both CESC and cES cells after HMBA treatment (F and ARAP3, LOC421054, LOC422654 and CDKN2A found down-regulated in CESC, but up-regulated in cES cells after HMBA treatment (G). Expression was also analysed in CEF and DF1 fibroblasts, in LMH, and in ESCDL1 cells. Expression was taken to be 1 in cES as a reference, and two independent samples were run, each in triplicate. Error bars indicate SD. (TIF) [file pone.0175259.s004.tif]

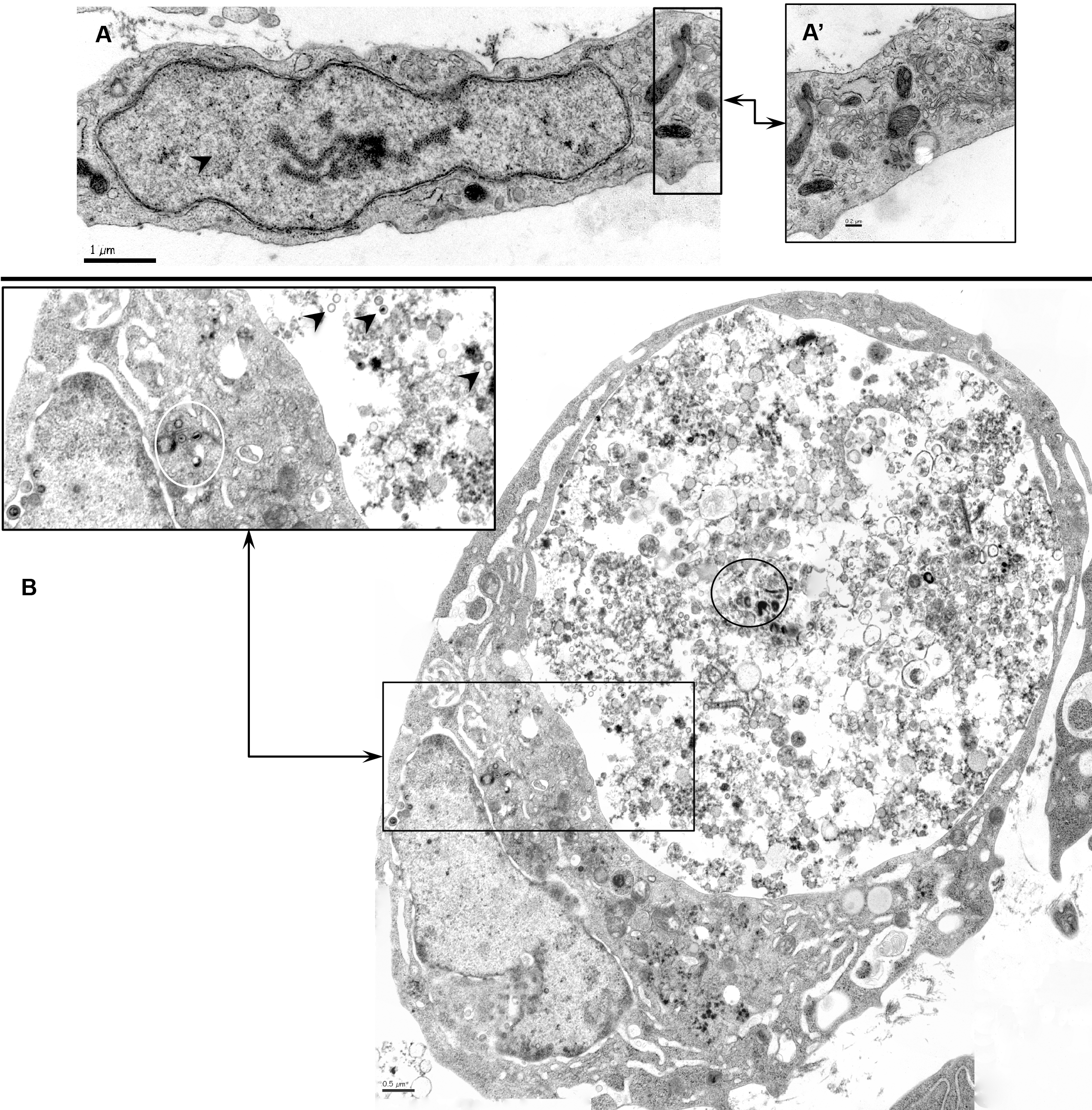

Supplement: S5 Fig — A and A’) Overview of a non-infected cell. Due to the elongated shape of the cells only the nucleus region is presented to illustrate the characteristic morphology of these cells. A’ inlet shows a magnification of the boxed zone in A. B) Overview of a highly infected cell undergoing vacuolization. Intranuclear A/B/C capsids are present and a primary enveloped particle can be seen in the nuclear membrane lamina (magnification of the boxed area). Intracytoplasmic capsids are encircled in white in the boxed area. Numerous A and C capsids can be seen in the vacuole (magnification of the boxed area, black arrowheads) in which electron dense material reminiscent of viral tegument particle (L particles in HSV-1) is also present (black circle). (TIF) [file pone.0175259.s005.tif]
