# Supplementary material for: ESCDL-1, a new cell line derived from chicken embryonic stem cells, supports efficient replication of Mardiviruses
Source: PLoS One. 2017 Apr 13;12(4):e0175259. doi: 10.1371/journal.pone.0175259 (PMC5391029; doi:10.1371/journal.pone.0175259)
Supplement: S1 Table — (DOCX) [file pone.0175259.s009.docx]

**S1 Table. List of oligonucleotides used in quantitative real time PCR analyses.**

| QRT PCR primers | | |
| --- | --- | --- |
|  | Forward | Reverse |
| **AK1** | ccccacgctgctgctctacg | ctcgttgtcgtccacccgcc |
| **ARAP3** | gaaccgcatcttcgaactcatc | tccatccactcccgtttctc |
| **ATP12A** | gagaccaggaacattgcattctac | caatgggtgttctctcgtttcc |
| **CPM** | ggagctgtcgtgttgtaaatatcc | tcacctgacctttgactcctaag |
| **CDKN2A** | tcgcgagcctgcaaggaagc | cgagcgccgatgatgcgtct |
| **DPT** | ccagacctgctcaaacaatgg | gccctgggtattctgttgttaac |
| **ECM2** | gtcagaaatttgctttaaccacacaag | tttatccaagccaaaggtgctattc |
| **FABP4** | ctggaagctcctttctagtgaaaac | gccatcttcctggtagcaaac |
| **HS6ST3** | tccaaagcgccaagaacaatc | gacgttgaactgggtgaatgg |
| **ID2** | gcccagcatcccgcagaaca | ggctgacgatggacgggtgc |
| **IGFBP2** | gagcgaggtcccctggagca | gggtccacgcaccagcactc |
| **IRF1** | gccatcttcctggtagcaaac | gacgttgaactgggtgaatgg |
| **KRT15** | tcgagactacagcaactactacaag | gcctggcgttgtcaatctc |
| **LCN8** | agtgatgatggtgaagtctactactc | tgcaaagattactgcatagctcttg |
| **LGALS1** | ggcacgtggggtgaggagga | tgcccagccgattgggaaact |
| **LOC421054** | ctcatcatgggtattggcatcttc | gcaggttgatgatcttggtcttc |
| **LOC422654** | gaagtcatagccacgacgaaac | ctgtgcagcgctgttgag |
| **MGAT3** | gccacggcccctcaagttcc | cgcccaccagcagggaagtg |
| **NANOG** | tgcacaccaggcttacagcagtg | tgctgggtgttgcagcttgttc |
| **NAPEPLD** | gcttccgaccacctccgtgc | agcgcagctcactgccgaag |
| **OLFM3** | tgggcaggaaccaaccacgtt | gtgcaagcacccgcccagta |
| **SCD5** | gatgcggacccacacaatg | tttcttcccttttctatgacgtctttg |
| **SCNN1A** | gtattggcagtttggaatcctctac | gtttaggttgaggttgaggttgac |
| **TERG1L** | gagagaggggtttcagcattttc | actcttctcttatcctggtcttgac |
| **THBS2** | ccattcactgggcacggggc | cccgcatccgcctctgcaat |
| **THBS4** | ccagcccagccaatgccaca | gggggtgccaggctgagaga |
| **WISP1** | agcccaactgcaaatacaactg | tgagtttgtgcacatgggaatg |
