# Supplementary material for: ESCDL-1, a new cell line derived from chicken embryonic stem cells, supports efficient replication of Mardiviruses
Source: PLoS One. 2017 Apr 13;12(4):e0175259. doi: 10.1371/journal.pone.0175259 (PMC5391029; doi:10.1371/journal.pone.0175259)
Supplement: S2 Table — (DOCX) [file pone.0175259.s010.docx]

**S2 Table. Differential gene expression in HMBA treated vs non-treated cES cells compared to CESC.**

| Differential expression in HMBA treated vs non-treated cells | | | | | | |
| --- | --- | --- | --- | --- | --- | --- |
|  | | CESC | | cES | | |
|  | Condition | HMBA + vs HMBA — | HMBA + /Virus vs HMBA + | | HMBA + vs HMBA — | HMBA +/Virus vs HMBA + |
| **Gene ID** | **Total** | **1092** | **425** | | **548** | **18** |
|  | Up | 192 | 271 | | 159 | 18 |
|  | Down | 900 | 154 | | 389 | 0 |
| **Gene Symbol** | **Total** | **563** | **198** | | **266** | **9** |
|  | Up | 72 | 133 | | 66 | 9 |
|  | Down | 491 | 65 | | 200 | 0 |
