# Supplementary material for: ESCDL-1, a new cell line derived from chicken embryonic stem cells, supports efficient replication of Mardiviruses
Source: PLoS One. 2017 Apr 13;12(4):e0175259. doi: 10.1371/journal.pone.0175259 (PMC5391029; doi:10.1371/journal.pone.0175259)
Supplement: S3 Table — (DOCX) [file pone.0175259.s011.docx]

**S3 Table. Viral replication on ESCDL-1-Venus (EV7) compared to the parental ESCDL-1.**

|  |  | Viruses | |
| --- | --- | --- | --- |
|  |  | vRB1BUL17mRFP/ESCDL-1/T2P3 | vRB1BUL17mRFP/EV7/T2P3* |
| Cells for titration | CESC | 5.67±1E+04 | 6.48±1E+04 |
|  | ESCDL-1 | 5.07±0.47E+04 | 7.86±0.6E+04 |
|  | EV7 | 4.57±0.45E+04 | 8.07±0.99E+04 |

- *Second passage on ESCDL-1-Venus/Clone7 (EV7)
- Titres were estimated from 2 independent titration experiments by recording plaque numbers in at least 4 wells per dilution
